# Efficacy of dietary supplements for treating knee osteoarthritis: a systematic review and network meta-analysis

**DOI:** 10.3389/fnut.2025.1556133

**Published:** 2025-03-07

**Authors:** Pupu Du, Asha Ajia, Zhi Xiang, Shang Zheng, Chenming Hu, Pingxi Wang

**Affiliations:** ^1^Dazhou Central Hospital, Dazhou, Sichuan, China; ^2^School of Medicine and Life Sciences, Chengdu University of Traditional Chinese Medicine, Chengdu, Sichuan, China; ^3^School of Clinical Medicine, North Sichuan Medical College, Nanchong, Sichuan, China; ^4^First School of Clinical Medicine, Shanxi Medical University, Taiyuan, Shanxi, China; ^5^Orthopaedics of Traditional Chinese Medicine, Dazhou Central Hospital, Dazhou, Sichuan, China

**Keywords:** knee osteoarthritis, dietary supplement, systematic review, network meta-analysis, efficacy

## Abstract

**Background:**

Knee osteoarthritis (KOA) stands as a prevalent clinical condition that frequently affects individuals. A growing body of research has highlighted the potential advantages of dietary supplements, including glucosamine and chondroitin, in the management of KOA.

**Purpose:**

This study aims to ascertain the most efficacious dietary supplement for KOA, with a specific focus on reducing pain, alleviating stiffness, and enhancing joint function.

**Methods:**

We conducted an exhaustive search of multiple databases, including PubMed, Web of Science, Embase, and the Cochrane Library, from inception to May 2023. We specifically focused on randomized controlled trials (RCTs) comparing various dietary supplements with the placebo group within the context of KOA. Assessment of outcomes among these groups relied on the Western Ontario and McMaster University Osteoarthritis Index (WOMAC), with weighted mean differences (WMDs) and associated 95% confidence intervals (CIs) computed. Network meta-analyses were employed to compare outcomes across different supplement groups in comparison with the placebo. The surface under the cumulative ranking curve (SUCRA) was utilized to rank these supplements.

**Results:**

Our comprehensive analysis included 22 studies with 2,777 participants in total. The outcomes from our network meta-analysis yielded the following key findings: To reduce the total WOMAC score, the top three interventions were E-OA-7, LParActin, and LcS. For reducing the WOMAC score of pain, the most effective interventions were Aflapin, NEM, and PFP. In addressing the reduction of the WOMAC score of stiffness, NEM, Aflapin, and MSM emerged as the optimal interventions. Finally, for diminishing the WOMAC score of physical function, the most effective interventions were E-OA-7, LParActin, and LcS.

**Conclusion:**

In comparison to the placebo, NEM (for stiffness), Aflapin (for pain), and E-OA-07 (for knee function and WOMAC total score) were discerned as the most effective interventions for the treatment of KOA.

**Clinical trial registration:**

https://www.crd.york.ac.uk/prospero/.

## Introduction

Knee osteoarthritis (KOA) represents a degenerative bone and joint condition that affects both men and women, primarily middle-aged and older adults. It stands as a major contributor to disability within this population. Its prevalence is on the rise as the population ages. Globally, an estimated 240 million persons are afflicted with symptomatic, activity-limiting OA (1, 2). OA of the knee and hip is a major cause of global disability (3), imposing substantial economic burdens. The estimated costs range from 1 to 2.5% of the gross domestic product (GDP) in Western countries (4). Wage losses due to OA amount to $65 billion, and direct medical costs exceed $100 billion (5). People with knee OA spend, on average, around $15,000 dollars (discounted) on the medical treatment of OA over their lifetimes (6).

Clinical guidelines currently advocate for paracetamol and non-steroidal anti-inflammatory drugs (NSAIDs) as the go-to treatments for OA (7–9). However, prolonged use of NSAIDs has been linked to undesirable side effects, including gastrointestinal complications, cardiovascular conditions, potential harm to the kidneys and liver (10, 11). Consequently, these drugs are not the preferred choice for long-term OA management. In tandem with conventional pharmaceutical options, nutritional interventions, such as nutraceuticals, have been increasingly used to manage and prevent KOA. Nutraceuticals, encompassing glycosaminoglycans and certain botanical extracts, have exhibited promise in reducing pain, improving function, and preserving joint space width (12). Approximately 30% of OA patients have incorporated supplements into their treatment regimen (13). An escalating body of research endorses the therapeutic effect of dietary supplements for KOA (14–16). Nevertheless, because of a lack of reproducibility in evidence and variabilities between dietary supplement manufactures (17), the optimal dietary supplement for this condition remains highly debatable. Hence, this up-to-date network meta-analysis aimed to compare the effectiveness of dietary supplements for patients with KOA. This method enables us to estimate the relative efficacy of all dietary supplements and subsequently rank them based on their observed impact.

## Methods

### Literature search strategy and eligibility criteria

This study was conducted in accordance with the Preferred Reporting Items for Systematic Reviews incorporating Network Meta-Analyses (PRISMA-NMA) guidelines and was registered on PROSPERO (CRD42023459251). Databases including PubMed, Embase, and the Cochrane Library were searched from inception to May 2023, for relevant studies published in English. Our search strategy involved the utilization of specific terms, including “Osteoarthritis, Knee” and “Dietary Supplements.” Detailed information on the literature search can be found in Supplementary Table S1. Furthermore, we conducted an exhaustive examination of the reference lists within pertinent reviews and eligible publications. We also explored ClinicalTrials.gov (US NIH) to identify completed studies that had not yet published their findings. After removing duplicate studies, two investigators independently evaluated the titles and abstracts of the remaining articles against predefined inclusion criteria and exclusion criteria. Inclusion criteria encompassed: (1) Study design: Randomized Controlled Trial (RCT); (2) Participants: individuals diagnosed with KOA; (3) Intervention and control groups involving a range of dietary supplements, including GSS, GS, MSM, A, GSS_A, NEM, MSM, VitD, CC, LART, HART, LcS, Garlic, LparActin, HparActin, non_animal CS, GS_CS, GCM, E_OA_07, AP, 5_Loxin, Aflapin, UC_II, GC, PFP, GCM; and (4) Outcomes: the primary outcome measure was the total Western Ontario and McMaster University Osteoarthritis Index (WOMAC) score, with secondary outcome measures encompassing WOMAC scores of pain, stiffness, and joint function. Exclusion criteria encompassed: (1) Editorials, reviews, previews, abstracts, letters, and nonhuman basic research; (2) Research in which data cannot be extracted; (3) Studies not written in English; (4) Duplicate publications that did not contain up-to-date data.

### Data collection and quality assessment

We systematically collected pertinent data, including the surname of the first author, publication year, country of origin, sample size, mean age, gender distribution, specific details regarding interventions and control groups, as well as the outcomes under investigation. The methodological quality of RCTs was evaluated utilizing the Cochrane risk-of-bias assessment tool, which evaluated six domains: random sequence generation, allocation concealment, blinding of participants, blinding of outcome assessors, handling of incomplete outcome data, and selective reporting. Each RCT was categorized as having a low, high, or unclear risk of bias in each of the six domains.

### Statistical analyses

In our statistical analyses, we conducted a Bayesian network meta-analysis employing R 4.2.1 software (R Foundation for Statistical Computing). Priori fuzzy fixed effects models were used to deal with data. Combined probabilities of each treatment being the best was derived from the results obtained from the Markov chain Monte Carlo (MCMC) simulations, where trajectory plots were employed to visualize the convergence of model parameters within a single chain and Brooks-Gelman-Rubin plots were utilized to assess the convergence of multiple parallel chains in MCMC simulations. Continuous outcomes were represented as standardized mean differences (SMD) along with corresponding 95% confidence intervals (CI). Heterogeneity was quantified using the *I*^2^ statistic. The surface under the cumulative ranking curve (SUCRA) was computed to estimate the probability of optimal intervention. Network diagrams were created using STATA 15.0 into which a specific pass-through macro command was loaded. These diagrams depicted dietary supplements as circles with edges connecting them representing comparisons between them, and the thickness of the circles was proportionate to the number of patients contributing to that specific comparison. Cumulative probability plots were drawn using the ggplot2 package.

## Results

### Literature search results

**Figure 1:**
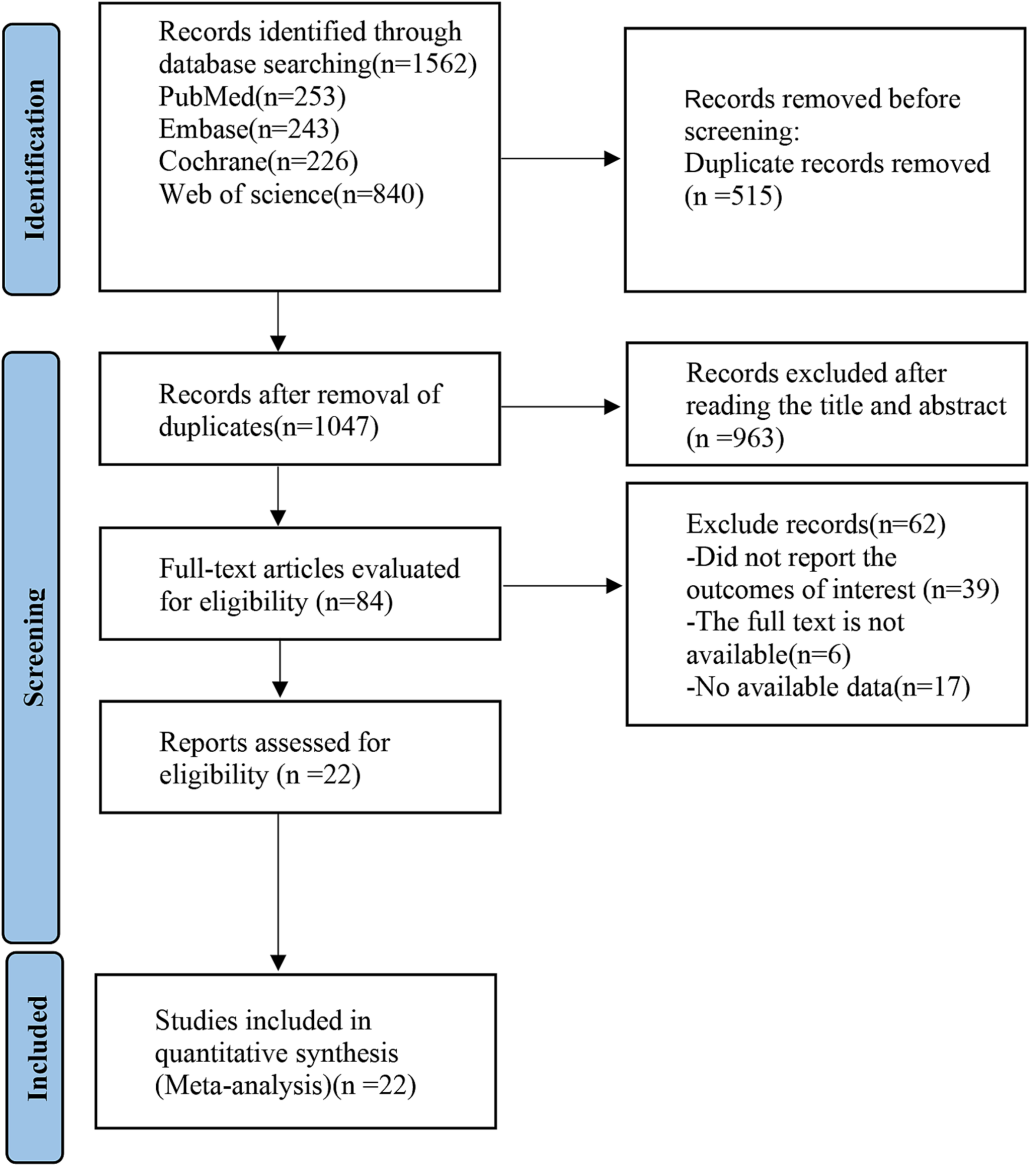
PRISMA flow diagram of the study process. PRISMA, Preferred Reporting Items for Systematic review and Meta-analysis.

According to the established retrieval strategy, 1,562 related articles were retrieved electronically, and 515 duplicate articles were excluded. After reading the tittle and abstract, 963 studies, such as conference summaries, abstracts, reviews, non-human studies, and non-English articles, were excluded. After carefully reading the full-text of the remaining studies, 62 articles that did not report the outcomes of interest, whose full text cannot be available and whose data could not be extracted were removed. Finally, 22 eligible studies were included, with 2,777 patients with KOA (Figure 1). Table 1 provides a comprehensive overview of the baseline demographic characteristics of these RCTs. To ensure a coherent analysis, we categorized the patients into 24 intervention groups based on the diverse treatments they received. Notably, within this selection, 17 studies reported outcomes using total WOMAC scores, while 21 studies provided WOMAC scores of pain, 20 studies focused on WOMAC scores of physical function, and 18 studies reported WOMAC scores of stiffness as outcome measures. It is noteworthy that, across these studies, the baseline characteristics of the patients, including age, gender distribution, and BMI, were generally comparable.

**Table 1:** Characteristics of included studies.

Study	Year	Country	Sample size	Gender (M/F)	Mean age (years)	BMI	Intervention	Outcome
McAlindon	2013	USA	VitD:73 Placebo:73	57/89	VitD:61.8 Placebo:63.0	VitD:30.5 ± 5.0 Placebo: 30.8 ± 6.4	VitD:2000 IU/daily	F1; F3
Pavelka	2002	Czech Republic	GSS:101 Placebo:101	45/157	GSS:63.5 Placebo:61.2	GSS:25.7 ± 1.8 Placebo:25.7 ± 2.1	GSS:1500 mg/daily	F1;F2;F3;F4
McAlindon	2004	UK	GS:101 Placebo:104	73/132	–	GS:31.0 ± 7.6 Placebo:34.1 ± 9.0	GS: 1500 mg/daily	F1;F2;F3;F4
Kim	2006	USA	MSM:21 Placebo:19	15/25	MSM:56.6 Placebo:55.6	–	MSM: 6 g/daily	F1;F2;F3;F4
Frestedt	2008	USA	GSS:15 A:20 GSS_A:19 Placebo:16	33/37	GSS:59.2 A:58.5 GSS + A: 60.3 Placebo:58.9	GSS:32.1 A:32.5 GSS + A: 30.5 Placebo:32.4	GSS:1500 mg/d A: 2400 mg/d GSS + A: 1500 mg + 2,400 mg	F1;F2;F3;F4
Kalman	2008	USA	AP:11 Placebo:9	9/11	AP:57.7 Placebo:54.6	–	AP:80 mg/ daily Placebo:16	F1; F2; F3
Frestedt	2009	USA	A:8 Placebo:14	15/7	A:62.5 Placebo:62.9	–	A:2400 mg/daily	F1;F2;F3;F4
Ruff	2009	USA	NEM:29 Placebo:31	–	–	–	NEM:500 mg/daily	F1;F2;F3;F4
Debbi	2011	Israel	MSM:25 Placebo:25	17/33	MSM:67.0 Placebo:71.0	MSM:31.4 ± 5.4 Placebo:28.6 ± 3.09	MSM:1125 mg/3/daily	F1;F2;F3;F4
Sanghi	2013	India	VitD:52 Placebo:51	37/66	VitD:53.24 Placebo:53.00	VitD:25.86 ± 2.46 Placebo:25.65 ± 2.58	VitD: 60000 IU/daily for 10 days, and 60,000 IU/monthly for 12 months	F1;F2;F3;F4
Panahi	2014	Iran	CC:27 Placebo:26	9/44	CC:57.32 Placebo:57.57	CC:28.75 ± 3.17 Placebo:29.64 ± 4.46	CC:500 mg/3/daily	F1;F2;F3;F4
Fransen	2015	Australia	GS:152 CS:151 GS_CS:151 Placebo:151	89/516	GS:61.2 CS:59.5 GS + CS:60.7 Placebo:60.6	GS:28.4 ± 4.7 CS:29.6 ± 5.4 GS + CS:28.8 ± 6.0 Placebo:29.1 ± 5.8	GS:1500 mg/daily CS:800 mg/daily GS + CS:1500 + 800 mg/daily	F1;F3
Lugo	2016	USA	UC_II:63 GC:65 Placebo:58	89/97	UC-II:53.5 GC:52.6 Placebo:53.1	UC-II:25.2 ± 0.37 GC:25.5 ± 0.40 Placebo:24.7 ± 0.40	UC-II:40 mg/daily GC:1500 + 1,200 mg/daily	F1; F2; F3;
Stebbings	2016	New Zealand	ART (low dose):14 ART (high dose):14 Placebo:14	22/20	ART (low dose):62.9 ART (high dose):66.2 Placebo:59.6	ART (low dose):31.6 ± 5.81 ART (high dose):30.3 ± 4.82 Placebo:28.4 ± 5.18	ART (low dose):150 mg/2/daily ART (high dose):300 mg/2/daily	F1;F2;F3;F4
Lei	2017	China	LcS:215 Placebo:218	192/241	LcS:66.5 Placebo:67.2	LcS:24.3 ± 2.5 Placebo:25.1 ± 3.1	LcS: 6 × 10*9 cfu/2/daily	F1;F2;F3;F4
Salimzadeh	2018	Iran	Garlic:39 Placebo:37	0/76	–	Garlic:31.6 ± 4 Placebo:31.7 ± 4	Garlic:1000 mg/daily	F1;F2;F3;F4
Hancke	2019	USA	ParActin (low dose):37 ParActin (high dose):35 Placebo:36	22/86	ParActin (low dose):53.2 ParActin (high dose):55.3 Placebo:55.7	ParActin (low dose):26.7 ± 1.8 ParActin (high dose):26.8 ± 1.6 Placebo:27.1 ± 1.7	ParActin (low dose):300 mg/daily ParActin (high dose):600 mg/daily	F1;F2;F3;F4
Rondanelli	2019	Italy	Non_animal CS:30 Placebo:30	22/38	non-animal CS:62.53 Placebo:62.77	non-animal CS:27.88 ± 3.60 Placebo:27.56 ± 3.38	non-animal CS:600 mg/daily	F4
Sengupta	2010	India	Aflapin:19 5_Loxin:19 Placebo:19	19/38	Aflapin:53.2 5_Loxin:51.6 Placebo:52.4	Aflapin:25.2 ± 3.0 5-Loxin:25.1 ± 3.8 Placebo:25.3 ± 4.4	Aflapin:100 mg/daily 5-Loxin: 100 mg/daily	F1;F2; F3
Lubis	2017	Indonesia	GCM:50 GS + CS:49 Placebo:48	56/91	GCM:58.3 GS + CS:60.9 Placebo:62.8	–	GCM:1500 + 1,200 + 500 mg/daily GS + CS:1500 + 1,200 mg/daily	F4
Srivastava	2019	India	E_OA_07:21 Placebo:23	14/30	E-OA-07:53 Placebo:52.43	E-OA-07:27.15 ± 1.46 Placebo:27.60 ± 1.53	E-OA-07:1000 mg/daily	F1;F2;F3;F4
Hosseinzadeh-Attar	2020	Iran	Garlic:23 Placebo:25	0/48	–	–	Garlic:1000 mg/daily	F1;F2;F3;F4

F1, WOMAC pain; F2, WOMAC stiffness; F3, WOMAC function; F4, total WOMAC; VitD, Vitamin D; GSS, Glucosamine sulfate/sulfate; GS, glucosamine; MSM, Methylsulfonylmethane; A, Aquamin; CS, chondroitin sulfate; AP, Hyal-Joint; NEM, Natural Eggshell Membrane; CC, Curcuminoid; UC_II, Undenatured type II collagen; ART, *Artemisia annua* extract; GC, glucosamine hydrochloride plus chondroitin sulfate; LcS, probiotic *Lactobacillus casei* Shirota; ParActin, an andrographolide-containing supplement; Aflapin, a novel synergistic composition derived from *Boswellia serrata* gum resin; 5_Loxin, a novel *Boswellia serrata* extract; GCM, glucosamine-chondroitin sulfate-methylsulfonylmethane; E_OA_07, a novel blend *of* 7 herbal ingredients.

### Quality of included RCTs

**Figure 2:**
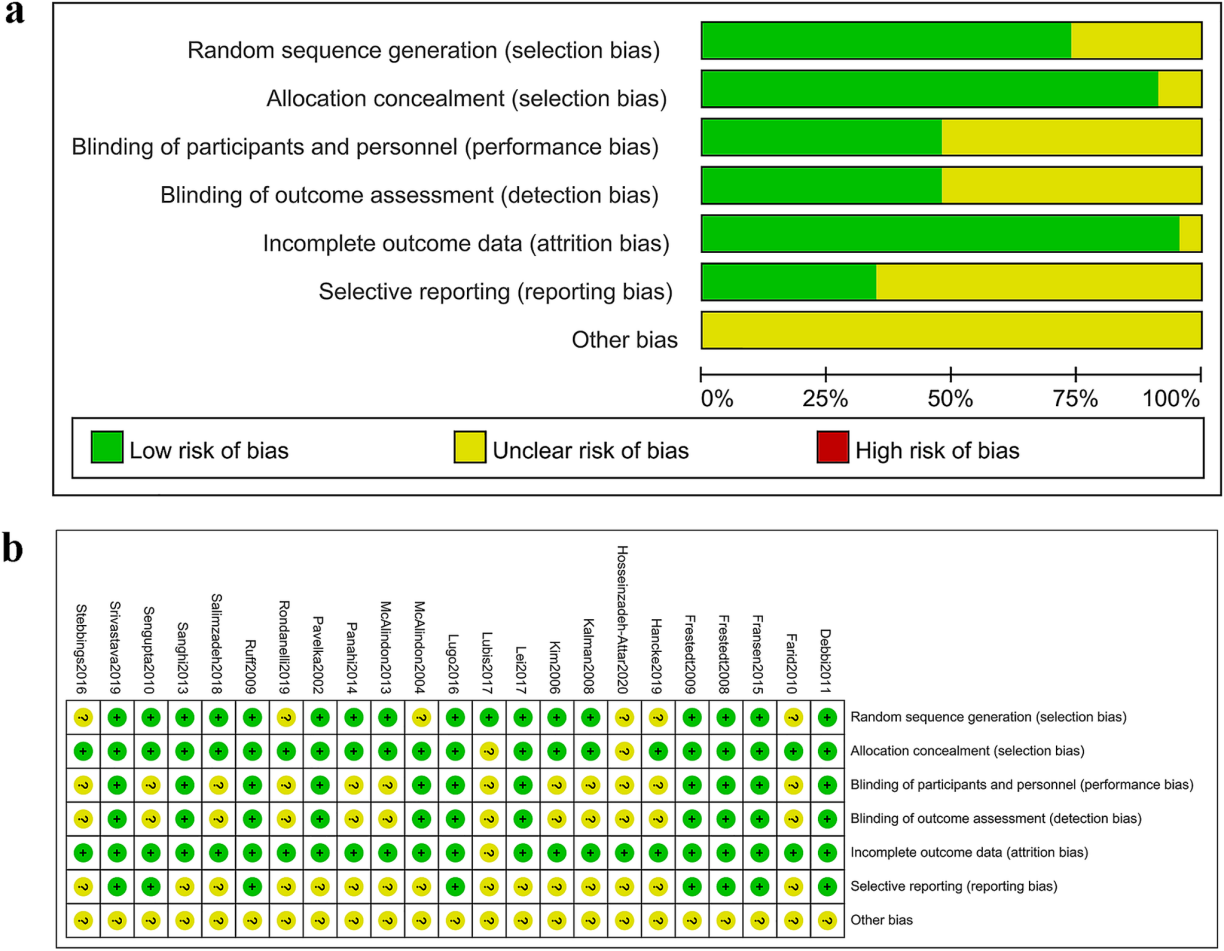
Risk of bias assessment. **(A)** Judgments of each bias item presented as percentages across all included studies. **(B)** Judgments of each bias item for each included study.

Figure 2A depicts the distribution of RCTs falling into low, medium, and high risk of bias within each evaluated domain. Figure 2B presents the results of the risk of bias assessment. In terms of the randomization process, 17 studies provided detailed information, including the generation of randomization sequences through methods such as computer numerical randomization or random number tables. The remaining six studies referred to randomization but omitted specific details, resulting in an “unclear” risk of bias rating. In the realm of allocation concealment, 21 studies demonstrated clarity and received a “low” risk of bias rating, while the remaining two studies omitted descriptions of allocation concealment, thus earning an “unclear” risk of bias rating. When it came to outcome evaluation, 11 studies reported implementing a blind methodology and thus received a “low” risk of bias rating, whereas the remaining two studies failed to mention such procedures and were consequently rated as having an “unclear” risk of bias. Information on patient withdrawals and reasons was provided by 20 studies, while the remaining three studies mentioned patient withdrawals without specifying the reasons, leading to an “unclear” risk of bias rating. None of the 49 studies provided detailed information on other potential sources of bias and were rated as having an “unclear” risk of bias.

### Total WOMAC score

**Figure 3:**
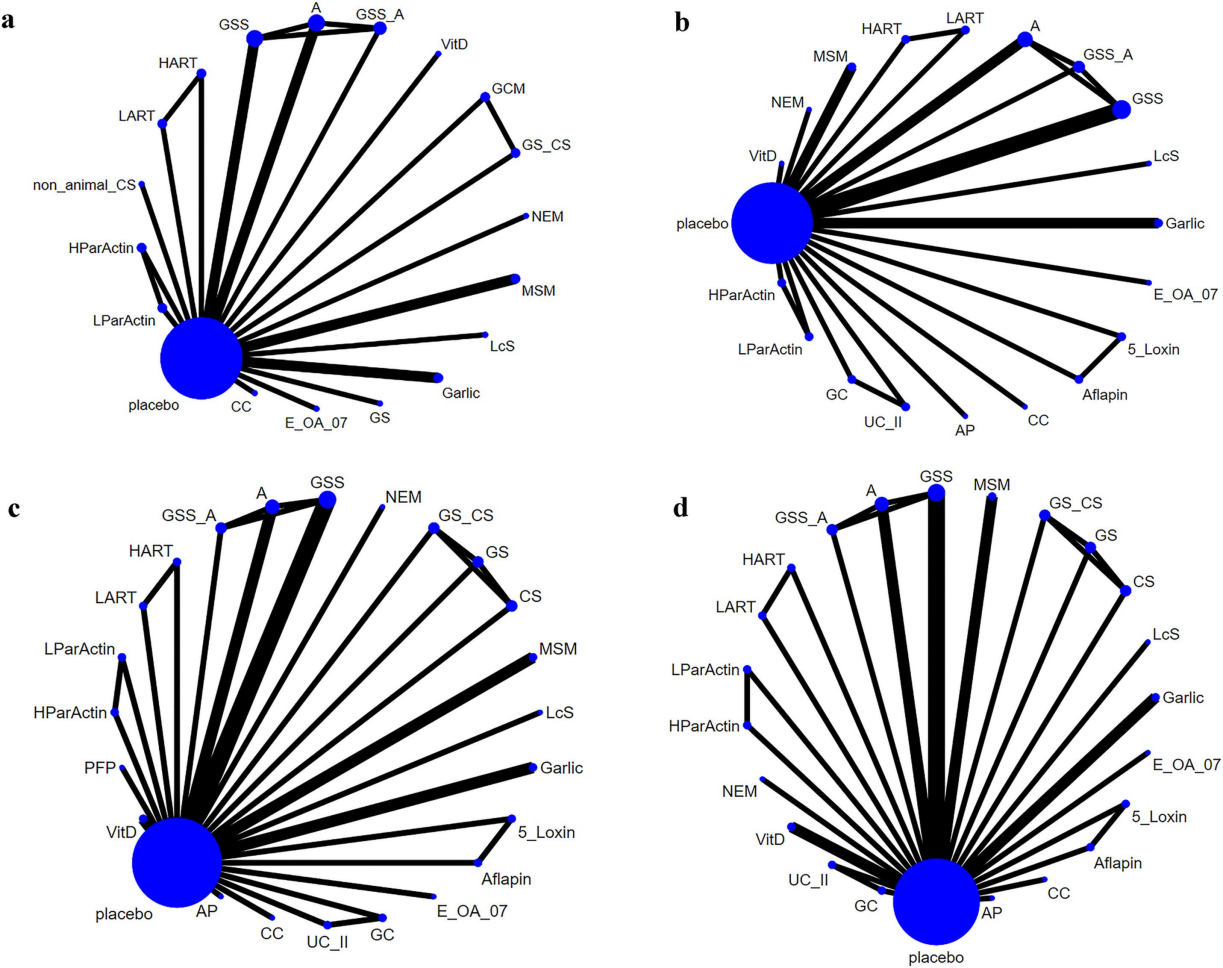
Network plot. **(A)** Total WOMAC score; **(B)** WOMAC scores of stiffness; **(C)** WOMAC scores of pain; **(D)** WOMAC scores of function.

**Figure 4:**
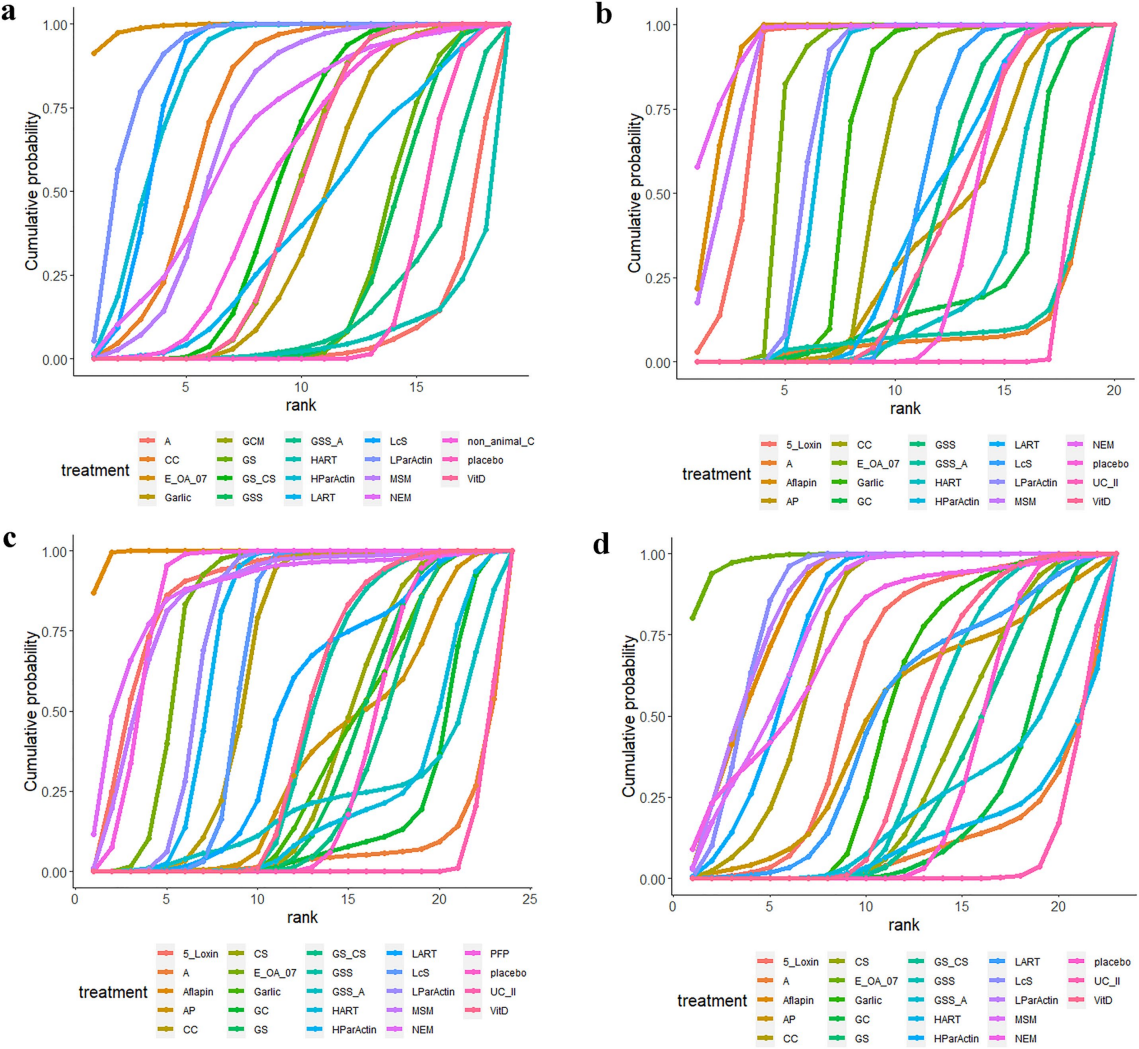
Cumulative probability graphs. **(A)** Total WOMAC score; **(B)** WOMAC scores of stiffness; **(C)** WOMAC scores of pain; **(D)** WOMAC scores of function.

Seventeen of the RCTs reported total WOMAC scores for 19 interventions. The network diagram (Figure 3A) revealed the formation of a closed loop. As a result, we conducted a local inconsistency test and the results (refer to Supplementary Figure S1) indicated no significant differences between GSS and A in direct comparisons, indirect comparisons, or network comparisons. Controls exhibited significantly worse total WOMAC scores when compared to all dietary supplements, except for HART, GSS_A, and A (WMD [95% CI]: CC, −15.6 [−23.5 to −7.61]; E_OA_07, −32.4 [−42.8 to −22]; Garlic, −5.55 [−10.8 to −0.31]; GCM, −7.16 [−12.1 to −2.28]; GS, −1.59 [−4.75 to 1.56]; GS_,CS −8.17 [−13.4 to −2.93]; GSS, −1.26 [−5.48 to 2.93]; HParActin, −19.8 [−26.8 to −12.9]; LART, −5.26 [−17.9 to 7.28]; LcS, −19.6 [−22.2 to −17.0]; LParActin, −22.6 [−29.5 to −15.8]; MSM, −14 [−22.6 to −5.33]; NEM, −13.4 [−28.0 to 1.16]; non_animal CS, −8.90 [−18.3 to 0.389]; VitD, −7.06 [−11.6 to −2.55]; Supplementary Figure S2). The SUCRA was highest for E-OA-07 (0.99), followed by LParActin (0.90), LcS (0.84), and lowest for HART (0.06) (Figure 4A; Supplementary Figure S3; Supplementary Table S2).

### WOMAC scores of stiffness

In 18 RCTs, WOMAC scores of stiffness were reported for 20 different interventions. Upon examining the network diagram (Figure 3B), we noticed the formation of a closed loop. Subsequently, a local inconsistency test was conducted and the results (Supplementary Figure S4) revealed no difference between GSS and A in direct comparisons, indirect comparisons, or network comparisons. Notably, control groups exhibited significantly worse WOMAC scores of stiffness, compared with all dietary supplements, except for GSS_A, GC, A, UC_II, and HART (WMD [95% CI]: 5_Loxin, −12.5 [−21.3 to −3.78]; Aflapin, −17.8 [−25 to −10.6]; AP, −0.000804 [−1.49 to1.49]; CC, −0.611 [−1.06 to −0.612]; E_OA_07, −2.53 [−3.45 to −1.60]; Garlic, −1.14 [−1.75 to −0.535]; GSS, −0.155 [−0.434 to 0.125]; HParActin, −1.72 [−2.25 to −1.19]; LART, −0.200 [−1.06 to 0.600]; LcS, −0.250 [−0.346 to −0.154]; LParActin, −1.83 [−2.36 to −1.30]; MSM, −16.2 [−26.8 to −5.60]; NEM, −21.5 [−36.2 to −6.90]; VitD, −0.0799 [−0.700 to 0.545]; Supplementary Figure S5). The SUCRA was highest for NEM (0.95), followed by Aflapin (0.93), MSM (0.91), and lowest for UC_II (0.06) (Figure 4B; Supplementary Figure S6; Supplementary Table 3).

### WOMAC scores of pain

In 21 RCTs, WOMAC scores of pain were reported for 24 different interventions. The network diagram (Figure 3C) exhibited the formation of a closed loop. After conducting a local inconsistency test, the results (Supplementary Figure S7) suggested no significant difference between GSS and A in direct comparisons, indirect comparisons, or network comparisons. Significantly worse WOMAC scores of pain were observed in control groups, compared with all dietary supplements, except for GC, GS_CS, GSS_A, A, UC_II, and HART (WMD [95% CI]: 5_Loxin, −10.9 [−20 to –-1.87]; Aflapin, −22.4 [−28.4 to −16.4]; AP, −0.106 [−3.45 to 3.25]; CC, −3.31 [−5.25 to −1.34]; GS, −0.0995 [−0.907 to 0.713]; GSS, −0.618 [−1.49 to 0.252]; HParActin, −4.46 [−5.61 to −3.32]; LART, −1.41 [−4.95 to 2.13]; LcS, −3.50 [−4.21 to −2.79]; LParActin, −4.80 [−6.03 to −3.57]; NEM, −13.2 [−27.0 to 0.761]; MSM, −10.1 [−19.1 to −1.07]; PFP, −9.60 [−13.5 to −5.70]; VitD, −0.686 [−1.58 to 0.203]; Supplementary Figure S8). The SUCRA was highest for Aflapin (0.99), followed by NEM (0.874), PFP (0.872), and lowest for UC_II (0.03) (Figure 4C; Supplementary Figure S9; Supplementary Table S4).

### WOMAC scores of function

In 20 RCTs, WOMAC scores of function were reported for 23 interventions. The network diagram (Figure 3D) also displayed the formation of a closed loop. After conducting a local inconsistency test, the results (Supplementary Figure S10) indicated no significant difference between GSS and A in direct comparisons, indirect comparisons, or network comparisons. Notably, control groups exhibited significantly worse WOMAC scores of function, compared with all dietary supplements, except for GC, GS, A, GS_CS, GSS_A, HART, and UC_II (WMD [95% CI]: 5_Loxin, −6.75 [−15.1 to −1.54]; Aflapin, −15.8 [−21.9 to −9.68]; AP, −4.26 [−17.1 to 8.51]; CC, −11.7 [−17.8 to −5.52]; CS, −0.414 [−3.34 to 2.55]; E_OA_07, −23.7 [−31.3 to −16.2]; Garlic, −2.91 [−6.79 to 0.985]; GSS, −1.11 [−3.20 to 0.988]; HParActin, −13.6 [−19.2 to −8.01]; LART, −3.81 [−13.5 to 5.94]; LcS, −15.8 [−17.8 to −13.8]; LParActin, −16.0 [−21.4 to −10.6]; MSM, −14.1 [−22.9 to −5.35]; NEM, −12.6 [−27.9 to 2.54]; VitD, −1.57 [−3.95 to 0.832]; Supplementary Figure S11). The SUCRA was found to be the highest for Aflapin (0.99), followed by E-OA-07 (0.98), followed by LParActin (0.859), LcS (0.858) and worst for UC_II (0.06) (Figure 4D; Supplementary Figure S12; Supplementary Table S5).

### Publication bias

**Figure 5:**
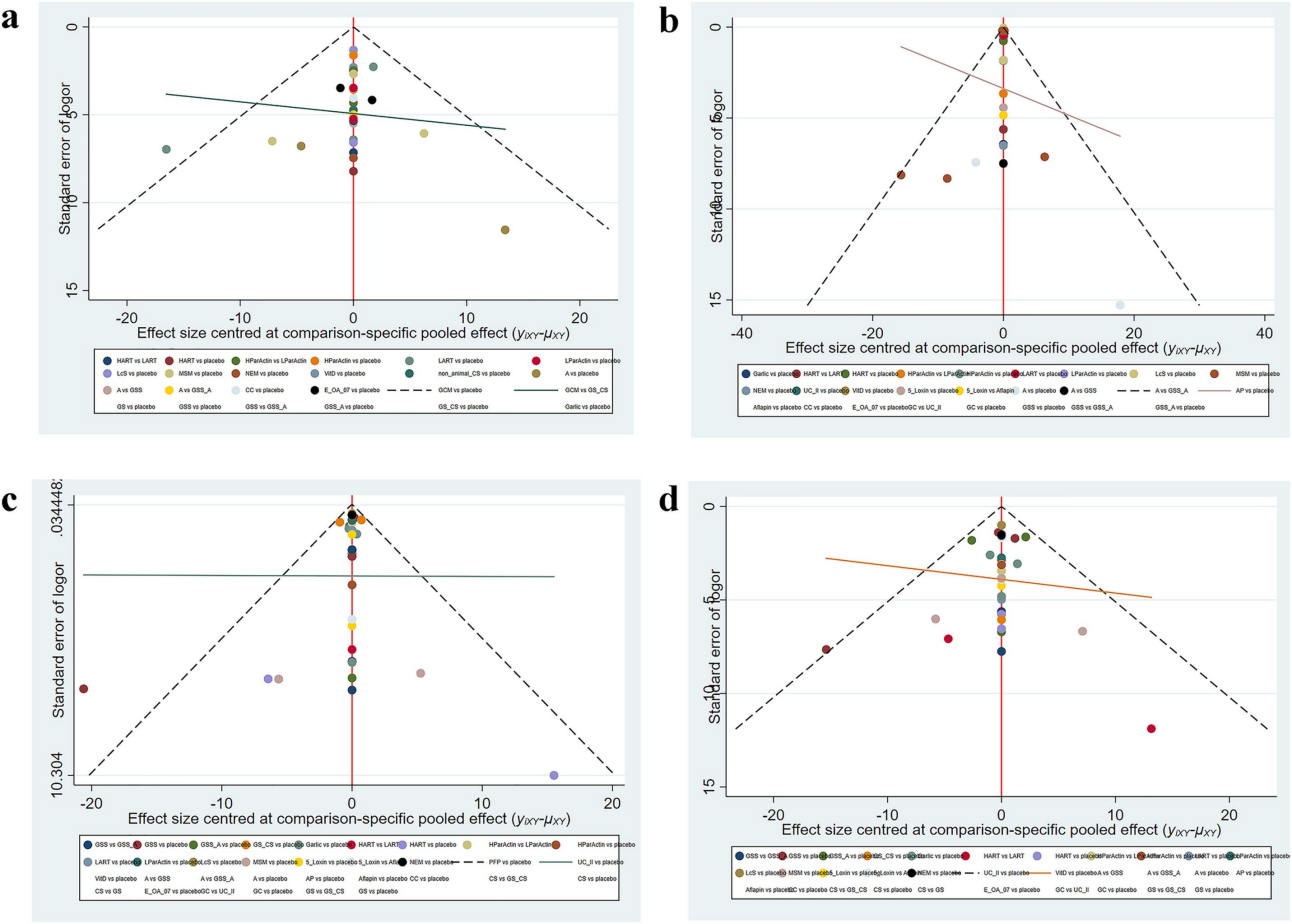
Funnel plot **(A)** Total WOMAC score; **(B)** WOMAC scores of stiffness; **(C)** WOMAC scores of pain; **(D)** WOMAC scores of function.

We assessed publication bias using funnel plots for four outcome measures. The results (Figure 5) indicated a slight possibility of the existence of publication bias regarding WOMAC total score, WOMAC stiffness score, WOMAC pain score, and WOMAC function score.

## Discussion

Our comprehensive analysis of 22 studies pinpointed standout interventions for KOA management in comparison to the placebo group. SUCRA values range from 1 (the best) to 0 (the worst). SUCRA values denote the benefits and limitations of each dietary supplement for KOA. Notably, NEM showcased remarkable efficacy in mitigating stiffness, Aflapin took the lead in alleviating pain, while E-OA-07 excelled in enhancing knee function and the total WOMAC score.

When examining the results from the WOMAC subscale of pain, the effectiveness ranking unfolds as follows: Aflapin > NEM > PFP > 5_Loxin > MSM > E_OA_07 > LParActin > HParActin > LcS > CC > LART > VitD > GSS > CS > AP > Garlic > GS > placebo > GS_CS > GSS_A > HART > GC > A > UC_II. Aflapin, in particular, demonstrated remarkable effectiveness in alleviating pain (SUCRA, 99.4%; WMD vs. placebo, −22.41 [95% CI −28.45 to −16.40]).

Aflapin^®^ represents a novel synergistic composition derived from *Boswellia serrata* gum resin (Indian Patent Application No. 2229/CHE/2008). Aflapin contains *B. serrata* extract enriched in AKBA and a non-volatile oil portion of *B. Serrata* gum resin. Its therapeutic efficacy against OA can be attributed to its influence on cellular and molecular mechanisms associated with the disease. Previous research has illustrated several advantageous effects of Aflapin in comparison with 5-Loxin. Firstly, Aflapin demonstrates enhanced anti-inflammatory efficacy by suppressing the activity of the 5-lipoxygenase enzyme and reducing TNFα production. Secondly, it offers substantial protection against the damaging effects of IL-1 by promoting chondrocyte proliferation and increasing the synthesis of cartilage matrix components like collagen and glycosaminoglycans in primary chondrocytes. Lastly, Aflapin also inhibits the production of MMP3 production in human chondrocytes induced by TNFα (18, 19). Sengupta et al. (18) reported significant improvements in pain scores among patients with KOA patients receiving Aflapin.

As for improvements in the scores for the WOMAC stiffness subscale, the ranking of effectiveness was as follows: NEM > Aflapin > MSM > 5_Loxin > E_OA_07 > LParActin > HParActin > Garlic > CC > LcS > LART > GSS > AP > VitD > placebo > HART > GC > GSS_A > A > UC_II. NEM was found to be the most effective intervention in addressing stiffness (WOMAC-Stiffness: SUCRA, 95.8%; WMD, −27.51 [95% CI, −42.57 to −12.43]).

Natural Eggshell Membrane (NEM^®^) is a dietary supplement primarily containing fibrous proteins that provide support and elasticity to tissues and bioactive glycosaminoglycans like dermatan sulfate, essential for maintaining tissue integrity and regulating cellular processes. NEM^®^ has the potential to support joint health and alleviate OA-related pain and stiffness by providing vital anabolic elements frequently lacking in today’s typical Western diet and by addressing inflammation through immunomodulation, mainly through oral tolerance mechanisms (12). Danesch et al. (20) demonstrated that supplementation with NEM^®^ led to a substantial reduction in stiffness, both rapidly (30 days) and continuously (60 days).

Based on the results derived from the WOMAC function subscale and the total WOMAC score, the effectiveness ranking shapes up as follows: E_OA_07 > LParActin > LcS > Aflapin > MSM > HParActin > NEM > CC > 5_Loxin > Garlic > LART > AP > VitD > GSS > CS > GS_CS > GS > placebo > GSS_A > GC > HART > A > UC_II for WOMAC-Function, and E_OA_07 > LParActin > LcS > HParActin > CC > MSM > NEM > non_animal CS > GS_CS > GCM > VitD > Garlic > LART > GS > GSS > placebo > GSS_A > A > HART for the overall WOMAC score. E_OA_07 stood out as the most effective intervention for improving function (WOMAC-Function: SUCRA, 98.5%; WMD, −23.7 [95% CI, −32.28 to −16.18]) and WOMAC total score (WOMAC total score: SUCRA, 99.2%; WMD, −32.43 [95% CI, −42.92 to −21.85]).

E-OA-07 is a polyherbal preparation designed to manage KOA by incorporating seven herbal ingredients, namely Chopchini (*Smilax china*), Rasana (Pluchea lanceolata), Ashwagandha (*Withania somnifera*), Shunthi (*Zingiber officinale*), Shallaki (*Boswellia serrata*), Shyonaka (Oroxylum indicum), and Guggul (Commiphora mukul), which are renowned for their analgesic and anti-inflammatory properties (14). *Zingiber officinale*, also known as ginger, is hypothesized to exert its effects by inhibiting COX enzymes and reducing the synthesis of leukotrienes and prostaglandins. This action influences the mechanism responsible for pain perception and has also shown promise in alleviating exercise-induced muscle pain (21). Pluchea lanceolata, on the other hand, possesses not only anti-inflammatory properties but also noteworthy analgesic effects. In a preclinical study, it even outperformed drugs like ibuprofen in terms of its anti-inflammatory action (22). As a result, the rapid relief from pain and stiffness attributed to E-OA-07 can be attributed to the combined analgesic effect of these ingredients. Evidence has also demonstrated that E-OA-07 provided superior and long-lasting relief from joint pain and stiffness compared to a placebo (23).

Strengths of this review include adherence to PRISMA guidelines, prospective registration, and the utilization of Cochrane systematic evaluation to assess evidence quality, enhancing the reliability of our findings. One distinctive aspect of our review is its comprehensive scope. Unlike previous reviews that were limited to conducting meta-analyses without the incorporation of network meta-analysis (3), our approach allowed us to delve into a broader spectrum of supplements. This expansion enabled us to analyze and present pooled treatment effects for a wider range of supplements. This comprehensive coverage is of significant value to healthcare professionals, particularly physicians and sports and exercise medicine practitioners, as it equips them with extensive information for selecting suitable supplements within their clinical practice.

### Limitations

This study does have some limitations that warrant consideration. Firstly, the number of RCTs included in our analysis was relatively small, and some of them had limited sample sizes, which may cause a certain degree of publication bias. Secondly, the majority of the studies incorporated into our analysis were short-term RCTs, which consequently resulted in an imperfect follow-up process. Thus, dietary supplements have a long-term effect on KOA remains elusive. Additionally, our inclusion criteria were restricted to studies reported and published exclusively in English, potentially leading to information loss and increased heterogeneity in our findings. Moreover, due to a scarcity of collected data, side effects were not analyzed in this meta-analysis. Future studies with larger sample sizes are necessary to explore the long-term effects and side effects of dietary supplements in the treatment of KOA, so as to provide more references for clinical practice.

## Conclusion

This study aims to ascertain the most efficacious dietary supplement for KOA, especially for reducing pain, alleviating stiffness, and enhancing joint function. In comparison to a placebo, NEM (for addressing stiffness), Aflapin (for managing pain), and E-OA-07 (for enhancing knee function and WOMAC total score) emerged as the most effective interventions. In the treatment of KOA, benefits and limitations of different therapies, and conditions of the patient should be comprehensively considered. Due to the small sample size and other reasons, the interpretation of the results of this study should be cautious. Meanwhile, it is essential to acknowledge that further research is imperative. More RCTs with larger sample sizes and enhanced methodological quality are warranted to substantiate the efficacy of dietary supplements in the management of KOA.

## Data Availability

The original contributions presented in the study are included in the article/Supplementary material, further inquiries can be directed to the corresponding author.
