# Supplementary material for: Efficacy of dietary supplements for treating knee osteoarthritis: a systematic review and network meta-analysis
Source: Front Nutr. 2025 Mar 7;12:1556133. doi: 10.3389/fnut.2025.1556133 (PMC11925762; doi:10.3389/fnut.2025.1556133)
Supplement: Supplementary file 1 [file Table_1.docx]

# Supplementary materials

**Table S1** Search strategy

**1.Pubmed**

| Search number | Query |
| --- | --- |
| #1 | "Osteoarthritis, Knee"[Mesh] |
| #2 | ((((Osteoarthritis, Knee[Title/Abstract]) OR (Knee Osteoarthritides[Title/Abstract])) OR (Knee Osteoarthritis[Title/Abstract])) OR (Osteoarthritis of Knee[Title/Abstract])) OR (Osteoarthritis of the Knee[Title/Abstract]) |
| #3 | "Dietary Supplements"[Mesh] |
| #4 | (((((((((((((((((((Dietary Supplements[Title/Abstract]) OR (Dietary Supplement[Title/Abstract])) OR (Supplements, Dietary[Title/Abstract])) OR (Dietary Supplementations[Title/Abstract])) OR (Supplementations, Dietary[Title/Abstract])) OR (Food Supplementations[Title/Abstract])) OR (Food Supplements[Title/Abstract])) OR (Food Supplement[Title/Abstract])) OR (Supplement, Food[Title/Abstract])) OR (Supplements, Food[Title/Abstract])) OR (Nutraceuticals[Title/Abstract])) OR (Nutraceutical[Title/Abstract])) OR (Nutriceuticals[Title/Abstract])) OR (Nutriceutical[Title/Abstract])) OR (Neutraceuticals[Title/Abstract])) OR (Neutraceutical[Title/Abstract])) OR (Herbal Supplements[Title/Abstract])) OR (Herbal Supplement[Title/Abstract])) OR (Supplement, Herbal[Title/Abstract])) OR (Supplements, Herbal[Title/Abstract]) |
| #5 | (#1 OR #2) AND (#3 OR #4) |

**2.Cochrane**

| Search number | Query |
| --- | --- |
| #1 | MeSH descriptor: [Osteoarthritis, Knee] explode all trees |
| #2 | (Osteoarthritis, Knee):ti,ab,kw OR (Knee Osteoarthritides):ti,ab,kw OR (Knee Osteoarthritis):ti,ab,kw OR (Osteoarthritis of Knee):ti,ab,kw OR (Osteoarthritis of the Knee):ti,ab,kw |
| #3 | MeSH descriptor: [Dietary Supplements] explode all trees |
| #4 | (Dietary Supplements):ti,ab,kw OR (Dietary Supplement):ti,ab,kw OR (Supplements, Dietary):ti,ab,kw OR (Dietary Supplementations):ti,ab,kw OR (Supplementations, Dietary):ti,ab,kw |
| #5 | (Food Supplementations):ti,ab,kw OR (Food Supplements):ti,ab,kw OR (Food Supplement):ti,ab,kw OR (Supplement, Food):ti,ab,kw OR (Supplements, Food):ti,ab,kw |
| #6 | (Nutraceuticals):ti,ab,kw OR (Nutraceutical):ti,ab,kw OR (Nutriceuticals):ti,ab,kw OR (Nutriceutical):ti,ab,kw OR (Neutraceuticals):ti,ab,kw |
| #7 | (Neutraceutical):ti,ab,kw OR (Herbal Supplements):ti,ab,kw OR (Herbal Supplement):ti,ab,kw OR (Supplement, Herbal):ti,ab,kw OR (Supplements, Herbal):ti,ab,kw |
| #8 | #1 OR #2 |
| #9 | #3 OR #4 OR #5 OR #6 OR #7 |
| #10 | #8 AND #9 |

**3.Embase**

| Search number | Query |
| --- | --- |
| #1 | 'knee osteoarthritis'/exp |
| #2 | 'osteoarthritis, knee':ab,ti |
| #3 | 'knee osteoarthritides':ab,ti |
| #4 | 'knee osteoarthritis':ab,ti |
| #5 | 'osteoarthritis of knee':ab,ti |
| #6 | 'osteoarthritis of the knee':ab,ti |
| #7 | #1 OR #2 OR #3 OR #4 OR #5 OR #6 |
| #8 | 'dietary supplement'/exp |
| #9 | 'dietary supplements':ab,ti |
| #10 | 'dietary supplement':ab,ti |
| #11 | 'supplements, dietary':ab,ti |
| #12 | 'dietary supplementations':ab,ti |
| #13 | 'supplementations, dietary':ab,ti |
| #14 | 'food supplementations':ab,ti |
| #15 | 'food supplements':ab,ti |
| #16 | 'food supplement':ab,ti |
| #17 | 'supplement, food':ab,ti |
| #18 | 'supplements, food':ab,ti |
| #19 | 'nutraceuticals':ab,ti |
| #20 | 'nutraceutical':ab,ti |
| #21 | 'nutriceuticals':ab,ti |
| #22 | 'nutriceutical':ab,ti |
| #23 | 'neutraceuticals':ab,ti |
| #24 | 'neutraceutical':ab,ti |
| #25 | 'herbal supplements':ab,ti |
| #26 | 'herbal supplement':ab,ti |
| #27 | 'supplement, herbal':ab,ti |
| #28 | 'supplements, herbal':ab,ti |
| #29 | #8 OR #9 OR #10 OR #11 OR #12 OR #13 OR #14 OR #15 OR #16 OR #17 OR #18 OR #19 OR #20 OR #21 OR #22 OR #23 OR #24 OR #25 OR #26 OR #27 OR #28 |
| #30 | #7 AND #29 |

**4.Web of science**

| Search number | Query |
| --- | --- |
| #1 | TS=( Osteoarthritis, Knee) OR TS=(Knee Osteoarthritides) OR TS=(Knee Osteoarthritis) OR TS=(Osteoarthritis of Knee) OR TS=(Osteoarthritis of the Knee) |
| #2 | TS=(Dietary Supplements) OR TS=(Dietary Supplement) OR TS=(Supplements, Dietary) OR TS=(Dietary Supplementations) OR TS=(Supplementations, Dietary) OR TS=(Food Supplementations) OR TS=(Food Supplements) OR TS=(Food Supplement) OR TS=(Supplement, Food) OR TS=(Supplements, Food) OR TS=(Nutraceuticals) OR TS=(Nutraceutical) OR TS=(Nutriceuticals) OR TS=(Nutriceutical) OR TS=(Neutraceuticals) OR TS=(Neutraceutical) OR TS=(Herbal Supplements) OR TS=(Herbal Supplement) OR TS=(Supplement, Herbal) OR TS=(Supplements, Herbal) |
| #3 | #2 AND #1 |

**Table S2** Network meta-analysis of WOMAC scores of function for different interventions

|  | X5_Loxin | A | Aflapin | AP | CC | CS | E_OA_07 | Garlic | GC | GS |
| --- | --- | --- | --- | --- | --- | --- | --- | --- | --- | --- |
| 5_Loxin | 5_Loxin |  |  |  |  |  |  |  |  |  |
| A | -11.89 (-24.55, 0.73) | A |  |  |  |  |  |  |  |  |
| Aflapin | 8.96 (1.33, 16.62) | 20.86 (9.53, 32.21) | Aflapin |  |  |  |  |  |  |  |
| AP | -2.6 (-17.96, 12.77) | 9.28 (-6.67, 25.39) | -11.56 (-25.88, 2.65) | AP |  |  |  |  |  |  |
| CC | 4.94 (-5.4, 15.15) | 16.81 (5.42, 28.13) | -4.05 (-12.66, 4.55) | 7.53 (-6.7, 21.78) | CC |  |  |  |  |  |
| CS | -6.4 (-15.18, 2.3) | 5.49 (-4.54, 15.55) | -15.38 (-22.12, -8.61) | -3.81 (-16.96, 9.43) | -11.31 (-18.11, -4.57) | CS |  |  |  |  |
| E_OA_07 | 16.89 (5.75, 28.14) | 28.83 (16.64, 41.01) | 7.93 (-1.76, 17.66) | 19.51 (4.57, 34.54) | 11.97 (2.33, 21.75) | 23.31 (15.22, 31.41) | E_OA_07 |  |  |  |
| Garlic | -3.91 (-13.08, 5.18) | 7.98 (-2.37, 18.32) | -12.88 (-20.1, -5.66) | -1.32 (-14.71, 12.16) | -8.82 (-16.09, -1.58) | 2.49 (-2.35, 7.35) | -20.82 (-29.37, -12.34) | Garlic |  |  |
| GC | -8.27 (-17.23, 0.57) | 3.62 (-6.5, 13.71) | -17.25 (-24.16, -10.37) | -5.7 (-18.92, 7.64) | -13.2 (-20.15, -6.26) | -1.89 (-6.26, 2.52) | -25.19 (-33.46, -16.99) | -4.38 (-9.43, 0.7) | GC |  |
| GS | -6.8 (-15.66, 1.94) | 5.08 (-4.92, 15.13) | -15.79 (-22.55, -9.01) | -4.22 (-17.38, 9.05) | -11.72 (-18.52, -4.95) | -0.41 (-3.41, 2.6) | -23.71 (-31.82, -15.64) | -2.89 (-7.77, 1.99) | 1.48 (-2.95, 5.9) | GS |
| GS_CS | -6.8 (-15.65, 1.92) | 5.08 (-4.94, 15.16) | -15.78 (-22.55, -9) | -4.22 (-17.42, 9.03) | -11.72 (-18.52, -4.92) | -0.41 (-3.44, 2.63) | -23.72 (-31.81, -15.62) | -2.89 (-7.79, 2.02) | 1.47 (-2.96, 5.9) | 0 (-3.07, 3.06) |
| GSS | -5.68 (-14.23, 2.81) | 6.21 (-3.36, 15.77) | -14.66 (-21.08, -8.24) | -3.1 (-16.12, 9.98) | -10.6 (-17.08, -4.17) | 0.7 (-2.87, 4.32) | -22.59 (-30.45, -14.8) | -1.78 (-6.19, 2.61) | 2.59 (-1.3, 6.47) | 1.12 (-2.52, 4.74) |
| GSS_A | -8.84 (-21.23, 3.58) | 3.06 (-6.17, 12.25) | -17.8 (-28.84, -6.78) | -6.25 (-22.07, 9.67) | -13.77 (-24.82, -2.67) | -2.45 (-12.12, 7.31) | -25.74 (-37.7, -13.78) | -4.94 (-14.96, 5.11) | -0.55 (-10.34, 9.24) | -2.03 (-11.75, 7.73) |
| HART | -11.97 (-26.06, 2.07) | -0.04 (-14.9, 14.64) | -20.93 (-33.76, -8.12) | -9.35 (-26.42, 7.75) | -16.88 (-29.77, -3.97) | -5.55 (-17.26, 6.08) | -28.87 (-42.43, -15.27) | -8.01 (-20.01, 3.88) | -3.65 (-15.42, 8.01) | -5.14 (-16.88, 6.47) |
| HParActin | 6.82 (-3.28, 16.81) | 18.7 (7.59, 29.85) | -2.18 (-10.44, 6.11) | 9.4 (-4.71, 23.51) | 1.89 (-6.47, 10.18) | 13.2 (6.83, 19.55) | -10.11 (-19.5, -0.71) | 10.71 (3.87, 17.56) | 15.08 (8.54, 21.6) | 13.61 (7.22, 20.01) |
| LART | -2.98 (-15.73, 9.77) | 8.91 (-4.74, 22.56) | -11.95 (-23.38, -0.48) | -0.39 (-16.51, 15.75) | -7.91 (-19.42, 3.61) | 3.43 (-6.74, 13.6) | -19.89 (-32.2, -7.62) | 0.94 (-9.55, 11.44) | 5.32 (-4.93, 15.59) | 3.85 (-6.34, 14.01) |
| LcS | 9.01 (0.43, 17.46) | 20.89 (11.12, 30.68) | 0.02 (-6.36, 6.42) | 11.6 (-1.41, 24.69) | 4.08 (-2.37, 10.5) | 15.4 (11.86, 18.91) | -7.91 (-15.75, -0.13) | 12.9 (8.55, 17.27) | 17.28 (13.44, 21.13) | 15.81 (12.21, 19.37) |
| LParActin | 9.2 (-0.77, 19.03) | 21.09 (10.08, 32.03) | 0.21 (-7.91, 8.33) | 11.77 (-2.17, 25.79) | 4.27 (-3.9, 12.42) | 15.58 (9.43, 21.77) | -7.73 (-17.01, 1.56) | 13.1 (6.43, 19.77) | 17.47 (11.13, 23.82) | 15.98 (9.8, 22.18) |
| MSM | 7.28 (-4.73, 19.23) | 19.18 (6.23, 32.14) | -1.69 (-12.31, 8.95) | 9.88 (-5.67, 25.51) | 2.36 (-8.29, 13.09) | 13.67 (4.45, 22.92) | -9.64 (-21.17, 1.91) | 11.19 (1.66, 20.75) | 15.57 (6.2, 24.87) | 14.08 (4.84, 23.31) |
| NEM | 5.82 (-11.41, 23.16) | 17.73 (-0.16, 35.67) | -3.14 (-19.49, 13.23) | 8.46 (-11.55, 28.4) | 0.91 (-15.48, 17.29) | 12.2 (-3.18, 27.71) | -11.08 (-27.96, 5.86) | 9.73 (-5.87, 25.42) | 14.1 (-1.4, 29.68) | 12.62 (-2.8, 28.11) |
| placebo | -6.79 (-15.12, 1.41) | 5.1 (-4.49, 14.66) | -15.79 (-21.85, -9.71) | -4.21 (-17.08, 8.73) | -11.72 (-17.84, -5.61) | -0.41 (-3.31, 2.52) | -23.7 (-31.28, -16.18) | -2.89 (-6.76, 0.98) | 1.48 (-1.8, 4.76) | 0 (-2.98, 2.97) |
| UC_II | -11.98 (-20.92, -3.16) | -0.08 (-10.2, 10) | -20.96 (-27.85, -14.08) | -9.38 (-22.66, 3.92) | -16.92 (-23.83, -10) | -5.59 (-9.93, -1.23) | -28.9 (-37.16, -20.69) | -8.09 (-13.12, -3.06) | -3.71 (-6.85, -0.58) | -5.19 (-9.56, -0.81) |
| VitD | -5.23 (-13.92, 3.33) | 6.67 (-3.26, 16.53) | -14.22 (-20.72, -7.7) | -2.64 (-15.68, 10.51) | -10.17 (-16.74, -3.59) | 1.16 (-2.63, 4.93) | -22.13 (-30.08, -14.28) | -1.33 (-5.87, 3.23) | 3.05 (-1, 7.1) | 1.57 (-2.26, 5.4) |

**Table S2** Network meta-analysis of WOMAC scores of function for different interventions (continue)

|  | GS_CS | GSS | GSS_A | HART | HParActin | LART | LcS | LParActin | MSM | NEM | placebo | UC_II | VitD |
| --- | --- | --- | --- | --- | --- | --- | --- | --- | --- | --- | --- | --- | --- |
| GS_CS | GS_CS |  |  |  |  |  |  |  |  |  |  |  |  |
| GSS | 1.12 (-2.54, 4.76) | GSS |  |  |  |  |  |  |  |  |  |  |  |
| GSS_A | -2.03 (-11.72, 7.72) | -3.15 (-12.34, 6.1) | GSS_A |  |  |  |  |  |  |  |  |  |  |
| HART | -5.13 (-16.85, 6.5) | -6.24 (-17.76, 5.16) | -3.11 (-17.78, 11.39) | HART |  |  |  |  |  |  |  |  |  |
| HParActin | 13.61 (7.19, 19.98) | 12.5 (6.46, 18.51) | 15.65 (4.72, 26.47) | 18.75 (6.11, 31.39) | HParActin |  |  |  |  |  |  |  |  |
| LART | 3.83 (-6.35, 14.03) | 2.72 (-7.23, 12.68) | 5.87 (-7.53, 19.26) | 8.96 (-2.23, 20.26) | -9.77 (-21.07, 1.58) | LART |  |  |  |  |  |  |  |
| LcS | 15.81 (12.21, 19.39) | 14.69 (11.81, 17.57) | 17.84 (8.34, 27.26) | 20.94 (9.51, 32.45) | 2.19 (-3.78, 8.19) | 11.97 (2.03, 21.94) | LcS |  |  |  |  |  |  |
| LParActin | 15.99 (9.79, 22.21) | 14.88 (9.06, 20.71) | 18.03 (7.27, 28.69) | 21.14 (8.58, 33.66) | 2.39 (-4.37, 9.13) | 12.16 (0.99, 23.33) | 0.19 (-5.56, 5.96) | LParActin |  |  |  |  |  |
| MSM | 14.09 (4.84, 23.35) | 12.96 (3.95, 21.97) | 16.13 (3.38, 28.78) | 19.24 (4.99, 33.53) | 0.47 (-9.91, 10.91) | 10.26 (-2.88, 23.39) | -1.72 (-10.7, 7.26) | -1.93 (-12.16, 8.37) | MSM |  |  |  |  |
| NEM | 12.62 (-2.77, 28.13) | 11.51 (-3.79, 26.88) | 14.68 (-3.1, 32.5) | 17.78 (-1.13, 36.77) | -0.96 (-17.15, 15.22) | 8.81 (-9.21, 26.86) | -3.18 (-18.41, 12.22) | -3.36 (-19.49, 12.84) | -1.45 (-18.89, 16.08) | NEM |  |  |  |
| placebo | 0 (-2.99, 2.98) | -1.11 (-3.22, 0.98) | 2.04 (-7.24, 11.26) | 5.15 (-6.09, 16.46) | -13.6 (-19.23, -7.96) | -3.84 (-13.58, 5.91) | -15.8 (-17.79, -13.82) | -15.99 (-21.43, -10.57) | -14.08 (-22.83, -5.33) | -12.61 (-27.87, 2.5) | placebo |  |  |
| UC_II | -5.18 (-9.57, -0.79) | -6.31 (-10.12, -2.46) | -3.15 (-12.94, 6.63) | -0.06 (-11.73, 11.74) | -18.79 (-25.28, -12.32) | -9.02 (-19.25, 1.24) | -20.99 (-24.77, -17.21) | -21.17 (-27.48, -14.87) | -19.26 (-28.61, -9.97) | -17.83 (-33.37, -2.3) | -5.19 (-8.4, -1.97) | UC_II |  |
| VitD | 1.57 (-2.29, 5.4) | 0.45 (-2.72, 3.61) | 3.61 (-6, 13.12) | 6.7 (-4.79, 18.28) | -12.04 (-18.15, -5.92) | -2.27 (-12.31, 7.77) | -14.24 (-17.33, -11.13) | -14.42 (-20.36, -8.46) | -12.52 (-21.6, -3.44) | -11.07 (-26.46, 4.26) | 1.57 (-0.82, 3.95) | 6.76 (2.75, 10.76) | VitD |

**Table S3** Network meta-analysis of WOMAC scores of pain for different interventions

|  | X5_Loxin | A | Aflapin | AP | CC | CS | E_OA_07 | Garlic | GC | GS |
| --- | --- | --- | --- | --- | --- | --- | --- | --- | --- | --- |
| 5_Loxin | 5_Loxin |  |  |  |  |  |  |  |  |  |
| A | -18.39 (-31.19, -5.5) | A |  |  |  |  |  |  |  |  |
| Aflapin | 11.42 (2.87, 20.08) | 29.78 (18.87, 40.83) | Aflapin |  |  |  |  |  |  |  |
| AP | -10.91 (-20.55, -1.2) | 7.48 (-2.26, 17.27) | -22.31 (-29.19, -15.41) | AP |  |  |  |  |  |  |
| CC | -7.71 (-16.94, 1.68) | 10.66 (1.33, 20.07) | -19.11 (-25.44, -12.79) | 3.2 (-0.71, 7.08) | CC |  |  |  |  |  |
| CS | -10.8 (-19.88, -1.56) | 7.57 (-1.63, 16.78) | -22.21 (-28.29, -16.14) | 0.1 (-3.35, 3.53) | -3.09 (-5.22, -0.98) | CS |  |  |  |  |
| E_OA_07 | -4.85 (-14.14, 4.62) | 13.51 (4.11, 22.97) | -16.26 (-22.69, -9.82) | 6.05 (1.98, 10.09) | 2.85 (-0.14, 5.86) | 5.95 (3.55, 8.35) | E_OA_07 |  |  |  |
| Garlic | -10.87 (-19.98, -1.58) | 7.5 (-1.76, 16.81) | -22.28 (-28.47, -16.09) | 0.02 (-3.61, 3.66) | -3.16 (-5.58, -0.75) | -0.07 (-1.7, 1.56) | -6.02 (-8.68, -3.34) | Garlic |  |  |
| GC | -13.21 (-22.88, -3.42) | 5.16 (-4.56, 15) | -24.61 (-31.51, -17.73) | -2.3 (-7.06, 2.46) | -5.5 (-9.42, -1.57) | -2.4 (-5.9, 1.09) | -8.35 (-12.43, -4.26) | -2.33 (-6, 1.35) | GC |  |
| GS | -10.9 (-19.98, -1.66) | 7.47 (-1.71, 16.7) | -22.31 (-28.4, -16.23) | 0 (-3.45, 3.43) | -3.2 (-5.32, -1.08) | -0.1 (-0.92, 0.72) | -6.05 (-8.46, -3.64) | -0.03 (-1.66, 1.6) | 2.3 (-1.19, 5.79) | GS |
| GS_CS | -11.1 (-20.17, -1.85) | 7.27 (-1.89, 16.49) | -22.51 (-28.58, -16.44) | -0.2 (-3.66, 3.24) | -3.39 (-5.53, -1.28) | -0.3 (-1.12, 0.53) | -6.25 (-8.66, -3.84) | -0.23 (-1.86, 1.4) | 2.1 (-1.4, 5.6) | -0.2 (-1.03, 0.63) |
| GSS | -10.39 (-19.44, -1.12) | 7.99 (-1.15, 17.19) | -21.79 (-27.86, -15.71) | 0.52 (-2.95, 3.96) | -2.68 (-4.82, -0.54) | 0.42 (-0.77, 1.6) | -5.53 (-7.96, -3.11) | 0.49 (-1.17, 2.14) | 2.82 (-0.7, 6.32) | 0.52 (-0.67, 1.71) |
| GSS_A | -14.35 (-27.75, -0.84) | 4.02 (-6.54, 14.53) | -25.77 (-37.42, -14.16) | -3.45 (-13.96, 7.03) | -6.66 (-16.79, 3.49) | -3.56 (-13.55, 6.43) | -9.51 (-19.73, 0.7) | -3.49 (-13.54, 6.55) | -1.16 (-11.66, 9.37) | -3.46 (-13.45, 6.53) |
| HART | -12.61 (-22.49, -2.64) | 5.76 (-4.17, 15.75) | -24.03 (-31.25, -16.83) | -1.71 (-6.86, 3.44) | -4.91 (-9.3, -0.52) | -1.81 (-5.83, 2.18) | -7.76 (-12.31, -3.24) | -1.74 (-5.92, 2.43) | 0.58 (-4.6, 5.78) | -1.71 (-5.73, 2.3) |
| HParActin | -6.54 (-15.66, 2.72) | 11.83 (2.62, 21.08) | -17.94 (-24.07, -11.81) | 4.36 (0.81, 7.89) | 1.17 (-1.11, 3.45) | 4.26 (2.86, 5.67) | -1.68 (-4.22, 0.86) | 4.33 (2.52, 6.16) | 6.67 (3.08, 10.24) | 4.37 (2.96, 5.77) |
| LART | -9.61 (-19.29, 0.19) | 8.74 (-1.06, 18.65) | -21.02 (-28.05, -14.03) | 1.28 (-3.59, 6.18) | -1.92 (-5.96, 2.17) | 1.18 (-2.45, 4.82) | -4.76 (-8.97, -0.55) | 1.25 (-2.55, 5.08) | 3.58 (-1.3, 8.5) | 1.28 (-2.34, 4.95) |
| LcS | -7.51 (-16.57, 1.71) | 10.87 (1.7, 20.07) | -18.91 (-24.99, -12.86) | 3.4 (-0.03, 6.8) | 0.2 (-1.89, 2.28) | 3.3 (2.22, 4.37) | -2.65 (-5.03, -0.27) | 3.36 (1.78, 4.95) | 5.69 (2.23, 9.16) | 3.4 (2.32, 4.47) |
| LParActin | -6.21 (-15.34, 3.07) | 12.15 (2.95, 21.44) | -17.62 (-23.76, -11.48) | 4.69 (1.1, 8.26) | 1.5 (-0.82, 3.8) | 4.6 (3.12, 6.06) | -1.35 (-3.93, 1.23) | 4.66 (2.79, 6.54) | 6.99 (3.37, 10.62) | 4.69 (3.22, 6.17) |
| MSM | -0.89 (-13.65, 11.97) | 17.48 (4.61, 30.39) | -12.33 (-23.18, -1.39) | 10 (0.33, 19.64) | 6.8 (-2.45, 16.08) | 9.9 (0.84, 19.01) | 3.97 (-5.36, 13.31) | 9.97 (0.83, 19.14) | 12.3 (2.64, 22.01) | 10 (0.95, 19.11) |
| NEM | 2.25 (-14.33, 18.81) | 20.58 (4.07, 37.24) | -9.18 (-24.31, 5.94) | 13.13 (-1.08, 27.32) | 9.92 (-4.03, 23.92) | 13.01 (-0.78, 26.88) | 7.06 (-6.91, 21.12) | 13.08 (-0.78, 27.01) | 15.42 (1.22, 29.68) | 13.11 (-0.67, 27) |
| PFP | -1.4 (-11.26, 8.59) | 16.96 (7.04, 27) | -12.8 (-19.97, -5.63) | 9.5 (4.35, 14.66) | 6.31 (1.94, 10.67) | 9.4 (5.41, 13.38) | 3.45 (-1.08, 7.97) | 9.47 (5.32, 13.61) | 11.8 (6.63, 16.96) | 9.51 (5.52, 13.49) |
| placebo | -11.01 (-20.04, -1.8) | 7.37 (-1.77, 16.55) | -22.41 (-28.45, -16.4) | -0.11 (-3.46, 3.23) | -3.3 (-5.26, -1.34) | -0.2 (-1.01, 0.6) | -6.15 (-8.42, -3.89) | -0.14 (-1.55, 1.28) | 2.2 (-1.2, 5.6) | -0.1 (-0.92, 0.71) |
| UC_II | -18.01 (-27.67, -8.23) | 0.35 (-9.37, 10.17) | -29.41 (-36.32, -22.51) | -7.12 (-11.89, -2.3) | -10.3 (-14.27, -6.33) | -7.2 (-10.76, -3.69) | -13.15 (-17.27, -9.04) | -7.14 (-10.87, -3.42) | -4.81 (-8.18, -1.46) | -7.1 (-10.65, -3.58) |
| VitD | -10.32 (-19.41, -1.09) | 8.06 (-1.12, 17.29) | -21.73 (-27.82, -15.64) | 0.59 (-2.89, 4.04) | -2.61 (-4.77, -0.46) | 0.49 (-0.72, 1.68) | -5.47 (-7.9, -3.02) | 0.56 (-1.12, 2.23) | 2.89 (-0.64, 6.4) | 0.59 (-0.63, 1.79) |

**Table S3** Network meta-analysis of WOMAC scores of pain for different interventions (continue)

|  | GS_CS | GSS | GSS_A | HART | HParActin | LART | LcS | LParActin | MSM | NEM | PFP | placebo | UC_II | VitD |
| --- | --- | --- | --- | --- | --- | --- | --- | --- | --- | --- | --- | --- | --- | --- |
| GS_CS | GS_CS |  |  |  |  |  |  |  |  |  |  |  |  |  |
| GSS | 0.72 (-0.48, 1.91) | GSS |  |  |  |  |  |  |  |  |  |  |  |  |
| GSS_A | -3.26 (-13.25, 6.72) | -3.98 (-13.93, 5.97) | GSS_A |  |  |  |  |  |  |  |  |  |  |  |
| HART | -1.51 (-5.54, 2.49) | -2.23 (-6.25, 1.79) | 1.74 (-8.96, 12.43) | HART |  |  |  |  |  |  |  |  |  |  |
| HParActin | 4.56 (3.16, 5.97) | 3.85 (2.4, 5.28) | 7.83 (-2.19, 17.83) | 6.08 (1.99, 10.16) | HParActin |  |  |  |  |  |  |  |  |  |
| LART | 1.48 (-2.16, 5.12) | 0.76 (-2.88, 4.43) | 4.74 (-5.82, 15.27) | 3.01 (-0.72, 6.69) | -3.08 (-6.8, 0.65) | LART |  |  |  |  |  |  |  |  |
| LcS | 3.59 (2.51, 4.67) | 2.88 (1.75, 4) | 6.85 (-3.12, 16.86) | 5.11 (1.11, 9.1) | -0.97 (-2.32, 0.39) | 2.11 (-1.51, 5.72) | LcS |  |  |  |  |  |  |  |
| LParActin | 4.89 (3.42, 6.37) | 4.17 (2.67, 5.68) | 8.16 (-1.88, 18.16) | 6.41 (2.29, 10.52) | 0.33 (-1.07, 1.73) | 3.41 (-0.35, 7.14) | 1.3 (-0.13, 2.72) | LParActin |  |  |  |  |  |  |
| MSM | 10.2 (1.13, 19.32) | 9.49 (0.41, 18.59) | 13.48 (0.04, 26.92) | 11.71 (1.84, 21.62) | 5.65 (-3.47, 14.78) | 8.72 (-1, 18.48) | 6.61 (-2.46, 15.71) | 5.31 (-3.8, 14.45) | MSM |  |  |  |  |  |
| NEM | 13.31 (-0.46, 27.19) | 12.6 (-1.2, 26.48) | 16.55 (-0.44, 33.68) | 14.83 (0.47, 29.18) | 8.75 (-5.07, 22.67) | 11.83 (-2.39, 26.08) | 9.72 (-4.07, 23.6) | 8.42 (-5.42, 22.34) | 3.13 (-13.37, 19.67) | NEM |  |  |  |  |
| PFP | 9.7 (5.7, 13.68) | 8.98 (4.97, 12.99) | 12.96 (2.26, 23.66) | 11.21 (5.66, 16.75) | 5.13 (1.08, 9.2) | 8.22 (2.92, 13.48) | 6.11 (2.14, 10.08) | 4.8 (0.7, 8.89) | -0.5 (-10.36, 9.36) | -3.6 (-18.06, 10.73) | PFP |  |  |  |
| placebo | 0.09 (-0.72, 0.91) | -0.62 (-1.49, 0.25) | 3.35 (-6.59, 13.31) | 1.61 (-2.3, 5.53) | -4.47 (-5.61, -3.32) | -1.38 (-4.94, 2.14) | -3.5 (-4.21, -2.79) | -4.8 (-6.03, -3.57) | -10.11 (-19.18, -1.07) | -13.22 (-27.1, 0.54) | -9.6 (-13.51, -5.7) | placebo |  |  |
| UC_II | -6.91 (-10.45, -3.38) | -7.62 (-11.18, -4.09) | -3.65 (-14.18, 6.85) | -5.39 (-10.59, -0.18) | -11.47 (-15.1, -7.86) | -8.39 (-13.33, -3.49) | -10.5 (-14.03, -6.99) | -11.8 (-15.46, -8.15) | -17.1 (-26.81, -7.48) | -20.22 (-34.56, -6.01) | -16.61 (-21.81, -11.43) | -7 (-10.45, -3.57) | UC_II |  |
| VitD | 0.78 (-0.43, 1.99) | 0.07 (-1.19, 1.31) | 4.05 (-5.94, 14.03) | 2.29 (-1.72, 6.31) | -3.78 (-5.24, -2.32) | -0.7 (-4.37, 2.95) | -2.81 (-3.95, -1.67) | -4.11 (-5.62, -2.58) | -9.42 (-18.51, -0.35) | -12.53 (-26.42, 1.28) | -8.91 (-12.92, -4.91) | 0.69 (-0.21, 1.58) | 7.69 (4.14, 11.26) | VitD |

**Table S4** Network meta-analysis of WOMAC scores of stiffness for different interventions

|  | X5_Loxin | A | Aflapin | AP | CC | E_OA_07 | Garlic | GC | GSS | GSS_A |
| --- | --- | --- | --- | --- | --- | --- | --- | --- | --- | --- |
| 5_Loxin | 5_Loxin |  |  |  |  |  |  |  |  |  |
| A | -20.2 (-33.68, -6.72) | A |  |  |  |  |  |  |  |  |
| Aflapin | 5.3 (-4.15, 14.82) | 25.51 (12.96, 38.1) | Aflapin |  |  |  |  |  |  |  |
| AP | -12.49 (-21.29, -3.69) | 7.7 (-2.69, 18.08) | -17.8 (-25.13, -10.5) | AP |  |  |  |  |  |  |
| CC | -11.88 (-20.56, -3.2) | 8.31 (-1.99, 18.59) | -17.2 (-24.36, -10.01) | 0.61 (-0.94, 2.17) | CC |  |  |  |  |  |
| E_OA_07 | -9.96 (-18.67, -1.27) | 10.23 (-0.09, 20.57) | -15.28 (-22.48, -8.05) | 2.53 (0.78, 4.27) | 1.92 (0.89, 2.94) | E_OA_07 |  |  |  |  |
| Garlic | -11.35 (-20.04, -2.67) | 8.84 (-1.43, 19.14) | -16.67 (-23.84, -9.46) | 1.14 (-0.47, 2.75) | 0.53 (-0.22, 1.28) | -1.39 (-2.49, -0.28) | Garlic |  |  |  |
| GC | -14.1 (-23.46, -4.7) | 6.09 (-4.78, 17.04) | -19.41 (-27.44, -11.4) | -1.6 (-5.49, 2.25) | -2.22 (-5.82, 1.38) | -4.14 (-7.82, -0.45) | -2.75 (-6.37, 0.89) | GC |  |  |
| GSS | -12.33 (-21, -3.68) | 7.85 (-2.42, 18.14) | -17.65 (-24.81, -10.48) | 0.16 (-1.35, 1.66) | -0.45 (-0.98, 0.07) | -2.37 (-3.34, -1.41) | -0.99 (-1.65, -0.32) | 1.76 (-1.81, 5.35) | GSS |  |
| GSS_A | -20.1 (-34.04, -6.1) | 0.1 (-10.71, 10.99) | -25.39 (-38.5, -12.27) | -7.58 (-18.61, 3.46) | -8.19 (-19.11, 2.78) | -10.11 (-21.09, 0.88) | -8.72 (-19.66, 2.24) | -5.97 (-17.46, 5.56) | -7.74 (-18.66, 3.23) | GSS_A |
| HART | -13.1 (-21.83, -4.29) | 7.09 (-3.27, 17.48) | -18.41 (-25.69, -11.11) | -0.6 (-2.64, 1.42) | -1.21 (-2.67, 0.23) | -3.13 (-4.8, -1.48) | -1.75 (-3.26, -0.24) | 1 (-2.84, 4.84) | -0.76 (-2.17, 0.65) | 6.98 (-4.09, 17.99) |
| HParActin | -10.76 (-19.45, -2.11) | 9.42 (-0.86, 19.72) | -16.08 (-23.27, -8.88) | 1.72 (0.14, 3.3) | 1.11 (0.42, 1.8) | -0.81 (-1.87, 0.26) | 0.58 (-0.22, 1.38) | 3.32 (-0.28, 6.94) | 1.57 (0.97, 2.15) | 9.3 (-1.67, 20.23) |
| LART | -12.29 (-20.99, -3.58) | 7.9 (-2.42, 18.22) | -17.61 (-24.81, -10.37) | 0.2 (-1.51, 1.92) | -0.41 (-1.38, 0.55) | -2.33 (-3.59, -1.07) | -0.94 (-2, 0.1) | 1.81 (-1.86, 5.48) | 0.04 (-0.86, 0.95) | 7.78 (-3.21, 18.74) |
| LcS | -12.24 (-20.9, -3.59) | 7.95 (-2.33, 18.24) | -17.55 (-24.72, -10.38) | 0.25 (-1.24, 1.74) | -0.36 (-0.81, 0.09) | -2.28 (-3.21, -1.35) | -0.89 (-1.5, -0.28) | 1.86 (-1.71, 5.44) | 0.1 (-0.2, 0.39) | 7.83 (-3.11, 18.76) |
| LParActin | -10.66 (-19.33, -2) | 9.53 (-0.75, 19.82) | -15.98 (-23.17, -8.79) | 1.83 (0.25, 3.4) | 1.22 (0.53, 1.9) | -0.7 (-1.76, 0.36) | 0.69 (-0.11, 1.48) | 3.44 (-0.18, 7.05) | 1.67 (1.08, 2.27) | 9.41 (-1.57, 20.35) |
| MSM | 3.7 (-10.07, 17.31) | 23.88 (9.08, 38.66) | -1.63 (-14.47, 11.13) | 16.16 (5.47, 26.84) | 15.55 (4.95, 26.16) | 13.64 (3.01, 24.28) | 15.03 (4.41, 25.62) | 17.76 (6.58, 28.97) | 16.01 (5.39, 26.61) | 23.73 (8.58, 39.07) |
| NEM | 9 (-8.01, 25.93) | 29.21 (11.35, 47) | 3.7 (-12.65, 20.01) | 21.5 (6.77, 36.21) | 20.89 (6.26, 35.5) | 18.97 (4.31, 33.62) | 20.36 (5.72, 35) | 23.11 (8.02, 38.14) | 21.35 (6.73, 35.95) | 29.07 (10.79, 47.35) |
| placebo | -12.49 (-21.15, -3.84) | 7.7 (-2.57, 17.99) | -17.8 (-24.97, -10.64) | 0 (-1.48, 1.49) | -0.61 (-1.06, -0.17) | -2.53 (-3.46, -1.6) | -1.14 (-1.75, -0.53) | 1.61 (-1.96, 5.18) | -0.15 (-0.43, 0.13) | 7.58 (-3.37, 18.51) |
| UC_II | -18.49 (-27.88, -9.11) | 1.7 (-9.17, 12.62) | -23.81 (-31.85, -15.76) | -6 (-9.9, -2.11) | -6.61 (-10.25, -2.98) | -8.53 (-12.25, -4.81) | -7.14 (-10.8, -3.49) | -4.39 (-7.98, -0.83) | -6.15 (-9.77, -2.55) | 1.59 (-9.9, 13.1) |
| VitD | -12.41 (-21.11, -3.74) | 7.78 (-2.51, 18.1) | -17.73 (-24.91, -10.52) | 0.08 (-1.53, 1.69) | -0.53 (-1.29, 0.24) | -2.45 (-3.56, -1.34) | -1.06 (-1.93, -0.19) | 1.69 (-1.94, 5.32) | -0.08 (-0.76, 0.61) | 7.66 (-3.3, 18.6) |

**Table S4** Network meta-analysis of WOMAC scores of stiffness for different interventions (continue)

|  | HART | HParActin | LART | LcS | LParActin | MSM | NEM | placebo | UC_II | VitD |
| --- | --- | --- | --- | --- | --- | --- | --- | --- | --- | --- |
| HART | HART |  |  |  |  |  |  |  |  |  |
| HParActin | 2.32 (0.85, 3.79) | HParActin |  |  |  |  |  |  |  |  |
| LART | 0.8 (-0.65, 2.25) | -1.52 (-2.53, -0.51) | LART |  |  |  |  |  |  |  |
| LcS | 0.85 (-0.53, 2.24) | -1.47 (-2, -0.93) | 0.05 (-0.81, 0.92) | LcS |  |  |  |  |  |  |
| LParActin | 2.43 (0.96, 3.91) | 0.11 (-0.47, 0.68) | 1.63 (0.62, 2.63) | 1.58 (1.05, 2.11) | LParActin |  |  |  |  |  |
| MSM | 16.76 (6.07, 27.46) | 14.44 (3.81, 25.06) | 15.97 (5.33, 26.57) | 15.92 (5.3, 26.51) | 14.34 (3.7, 24.94) | MSM |  |  |  |  |
| NEM | 22.1 (7.43, 36.8) | 19.78 (5.15, 34.4) | 21.3 (6.65, 35.94) | 21.25 (6.63, 35.85) | 19.67 (5.04, 34.3) | 5.36 (-12.77, 23.41) | NEM |  |  |  |
| placebo | 0.6 (-0.77, 1.99) | -1.72 (-2.24, -1.19) | -0.2 (-1.06, 0.66) | -0.25 (-0.35, -0.15) | -1.83 (-2.35, -1.31) | -16.16 (-26.77, -5.56) | -21.5 (-36.11, -6.89) | placebo |  |  |
| UC_II | -5.39 (-9.27, -1.54) | -7.72 (-11.36, -4.08) | -6.2 (-9.91, -2.5) | -6.25 (-9.86, -2.65) | -7.83 (-11.48, -4.2) | -22.17 (-33.41, -10.95) | -27.51 (-42.57, -12.43) | -6 (-9.61, -2.4) | UC_II |  |
| VitD | 0.69 (-0.83, 2.2) | -1.64 (-2.46, -0.82) | -0.12 (-1.18, 0.95) | -0.17 (-0.8, 0.46) | -1.75 (-2.56, -0.94) | -16.08 (-26.7, -5.45) | -21.41 (-36.04, -6.8) | 0.08 (-0.54, 0.7) | 6.08 (2.42, 9.75) | VitD |

**Table S5** Network meta-analysis of total WOMAC scores for different interventions

|  | A | CC | E_OA_07 | Garlic | GCM | GS | GS_CS | GSS | GSS_A | HART |
| --- | --- | --- | --- | --- | --- | --- | --- | --- | --- | --- |
| A | A |  |  |  |  |  |  |  |  |  |
| CC | 20.83 (8.66, 33.16) | CC |  |  |  |  |  |  |  |  |
| E_OA_07 | 37.67 (23.5, 51.75) | 16.81 (3.58, 30.01) | E_OA_07 |  |  |  |  |  |  |  |
| Garlic | 10.79 (0.17, 21.41) | -10.06 (-19.56, -0.53) | -26.87 (-38.63, -15.14) | Garlic |  |  |  |  |  |  |
| GCM | 12.43 (1.92, 22.86) | -8.44 (-17.79, 0.89) | -25.26 (-36.87, -13.62) | 1.63 (-5.51, 8.71) | GCM |  |  |  |  |  |
| GS | 6.86 (-2.96, 16.57) | -14 (-22.57, -5.41) | -30.82 (-41.81, -19.78) | -3.95 (-10.06, 2.14) | -5.56 (-11.39, 0.22) | GS |  |  |  |  |
| GS_CS | 13.43 (2.78, 24.05) | -7.42 (-16.99, 2.07) | -24.27 (-36.02, -12.4) | 2.63 (-4.74, 9.98) | 1.01 (-3.83, 5.84) | 6.58 (0.48, 12.66) | GS_CS |  |  |  |
| GSS | 6.52 (-2.65, 15.63) | -14.34 (-23.33, -5.36) | -31.17 (-42.5, -19.78) | -4.29 (-10.97, 2.42) | -5.91 (-12.34, 0.5) | -0.34 (-5.59, 4.92) | -6.92 (-13.59, -0.22) | GSS |  |  |
| GSS_A | 3.28 (-6.25, 12.83) | -17.55 (-29.97, -5.29) | -34.39 (-48.52, -20.25) | -7.5 (-18.28, 3.25) | -9.11 (-19.72, 1.41) | -3.55 (-13.53, 6.37) | -10.12 (-20.94, 0.64) | -3.22 (-12.39, 5.96) | GSS_A |  |
| HART | -3.65 (-22.18, 14.98) | -24.49 (-42.38, -6.49) | -41.26 (-60.47, -22.13) | -14.43 (-31.41, 2.53) | -16.04 (-32.86, 0.79) | -10.48 (-26.8, 5.98) | -17.04 (-33.99, -0.12) | -10.17 (-26.69, 6.58) | -6.93 (-25.48, 11.85) | HART |
| HParActin | 25.09 (13.45, 36.59) | 4.21 (-6.41, 14.74) | -12.6 (-25.21, 0.03) | 14.26 (5.54, 22.97) | 12.65 (4.18, 21.1) | 18.22 (10.56, 25.88) | 11.64 (2.97, 20.31) | 18.55 (10.47, 26.63) | 21.77 (10.09, 33.43) | 28.68 (11.23, 46.18) |
| LART | 10.53 (-5.16, 26.17) | -10.33 (-25.19, 4.6) | -27.12 (-43.54, -10.74) | -0.26 (-13.87, 13.37) | -1.89 (-15.37, 11.56) | 3.67 (-9.24, 16.7) | -2.91 (-16.53, 10.66) | 4.01 (-9.25, 17.28) | 7.22 (-8.52, 22.93) | 14.15 (0.09, 28.22) |
| LcS | 24.86 (15.22, 34.44) | 4.01 (-4.38, 12.35) | -12.84 (-23.63, -1.93) | 14.06 (8.23, 19.85) | 12.44 (6.92, 17.92) | 18 (13.9, 22.1) | 11.43 (5.58, 17.23) | 18.34 (13.4, 23.28) | 21.55 (11.82, 31.37) | 28.48 (12.11, 44.77) |
| LParActin | 27.88 (16.4, 39.38) | 7.04 (-3.45, 17.55) | -9.76 (-22.33, 2.78) | 17.09 (8.48, 25.63) | 15.47 (7.1, 23.84) | 21.04 (13.53, 28.56) | 14.46 (5.87, 23.05) | 21.38 (13.37, 29.37) | 24.59 (13, 36.18) | 31.52 (13.99, 48.96) |
| MSM | 19.23 (6.52, 31.95) | -1.62 (-13.42, 10.19) | -18.44 (-32.09, -4.79) | 8.46 (-1.71, 18.53) | 6.81 (-3.13, 16.74) | 12.39 (3.14, 21.66) | 5.81 (-4.29, 15.91) | 12.75 (3.07, 22.37) | 15.95 (3.17, 28.79) | 22.89 (4.57, 41.15) |
| NEM | 18.76 (1.44, 36.02) | -2.07 (-18.85, 14.49) | -18.92 (-36.93, -0.9) | 7.96 (-7.54, 23.45) | 6.33 (-9.03, 21.71) | 11.91 (-3.11, 26.86) | 5.36 (-10.21, 20.84) | 12.25 (-2.96, 27.4) | 15.46 (-1.88, 32.79) | 22.41 (0.57, 44.13) |
| non_animal_CS | 14.19 (1.04, 27.33) | -6.65 (-18.83, 5.56) | -23.47 (-37.52, -9.34) | 3.42 (-7.22, 14.02) | 1.79 (-8.7, 12.23) | 7.37 (-2.47, 17.15) | 0.79 (-9.88, 11.41) | 7.69 (-2.53, 17.86) | 10.91 (-2.35, 24.11) | 17.83 (-0.76, 36.37) |
| placebo | 5.25 (-4.03, 14.48) | -15.61 (-23.57, -7.64) | -32.43 (-42.92, -21.85) | -5.55 (-10.77, -0.33) | -7.17 (-12.03, -2.33) | -1.6 (-4.77, 1.56) | -8.17 (-13.39, -3) | -1.26 (-5.47, 2.93) | 1.96 (-7.46, 11.46) | 8.88 (-7.25, 24.99) |
| VitD | 12.31 (2.02, 22.64) | -8.54 (-17.7, 0.63) | -25.38 (-36.77, -13.89) | 1.51 (-5.38, 8.42) | -0.11 (-6.73, 6.49) | 5.46 (-0.04, 10.94) | -1.11 (-8.03, 5.76) | 5.8 (-0.34, 11.96) | 9.02 (-1.4, 19.46) | 15.94 (-0.8, 32.65) |

**Table S5** Network meta-analysis of total WOMAC scores for different interventions (Continued)

|  | HParActin | LART | LcS | LParActin | MSM | NEM | non_animal_CS | placebo | VitD |
| --- | --- | --- | --- | --- | --- | --- | --- | --- | --- |
| HParActin | HParActin |  |  |  |  |  |  |  |  |
| LART | -14.55 (-28.85, -0.21) | LART |  |  |  |  |  |  |  |
| LcS | -0.21 (-7.62, 7.18) | 14.33 (1.45, 27.12) | LcS |  |  |  |  |  |  |
| LParActin | 2.83 (-5.65, 11.29) | 17.37 (3.16, 31.66) | 3.02 (-4.26, 10.33) | LParActin |  |  |  |  |  |
| MSM | -5.84 (-17, 5.36) | 8.73 (-6.57, 23.95) | -5.6 (-14.67, 3.44) | -8.65 (-19.72, 2.41) | MSM |  |  |  |  |
| NEM | -6.31 (-22.43, 9.89) | 8.23 (-11.08, 27.54) | -6.11 (-20.95, 8.72) | -9.15 (-25.27, 6.95) | -0.48 (-17.46, 16.47) | NEM |  |  |  |
| non_animal_CS | -10.86 (-22.48, 0.69) | 3.68 (-12, 19.24) | -10.64 (-20.28, -1.05) | -13.69 (-25.21, -2.18) | -5.04 (-17.79, 7.75) | -4.54 (-21.86, 12.76) | non_animal_CS |  |  |
| placebo | -19.81 (-26.79, -12.87) | -5.26 (-17.9, 7.27) | -19.6 (-22.19, -17.02) | -22.63 (-29.47, -15.85) | -13.99 (-22.68, -5.31) | -13.49 (-28.09, 1.11) | -8.97 (-18.22, 0.36) | placebo |  |
| VitD | -12.75 (-21.03, -4.5) | 1.78 (-11.59, 15.12) | -12.54 (-17.73, -7.36) | -15.58 (-23.75, -7.42) | -6.92 (-16.67, 2.83) | -6.45 (-21.71, 8.85) | -1.88 (-12.21, 8.45) | 7.06 (2.56, 11.55) | VitD |


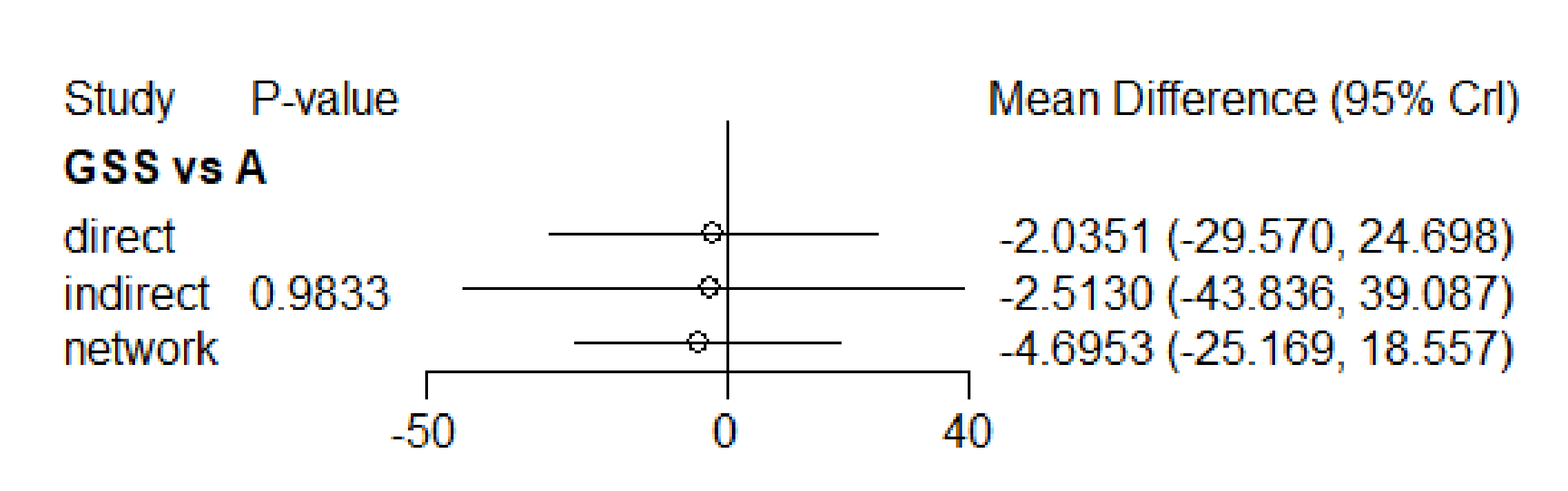


**Supplementary Figure 1 Node splitting diagram of total WOMAC score**

**
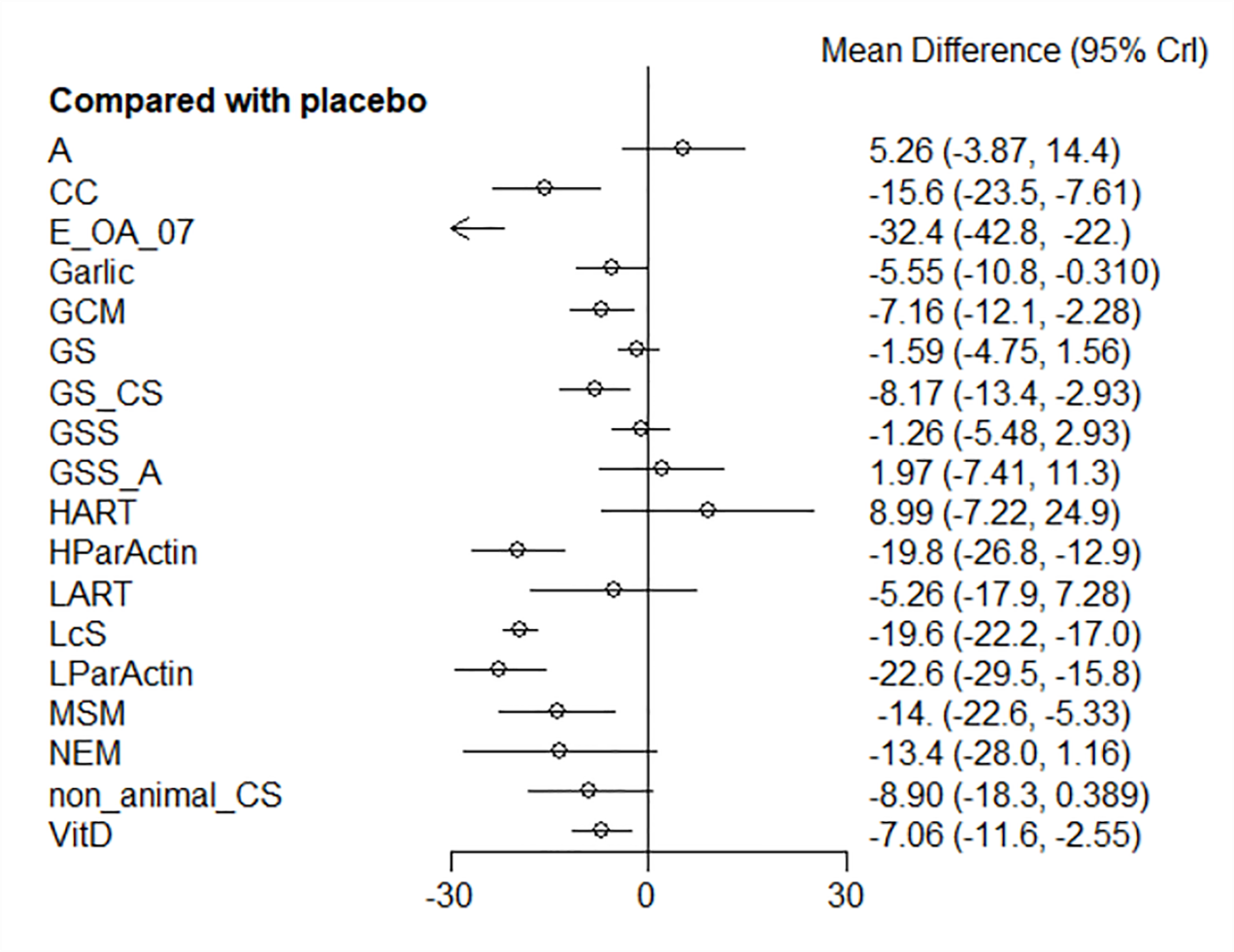
**

**Supplementary Figure 2 Forest plots of total WOMAC score**

**
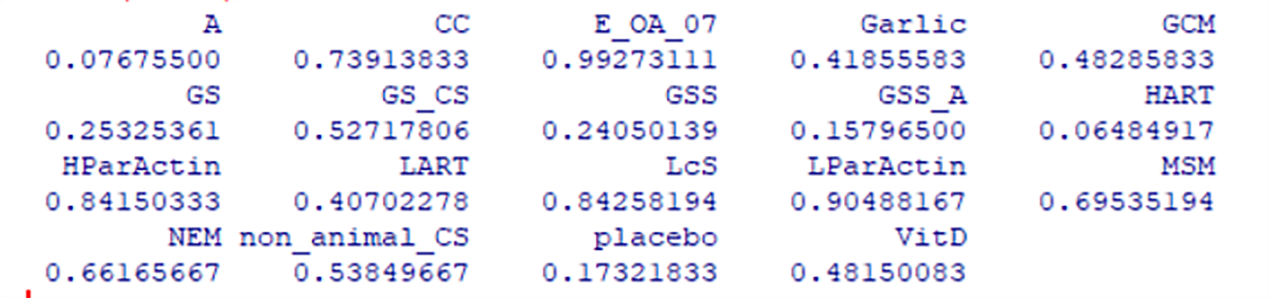
**

**Supplementary Figure 3 SUCRA of total WOMAC score**

**
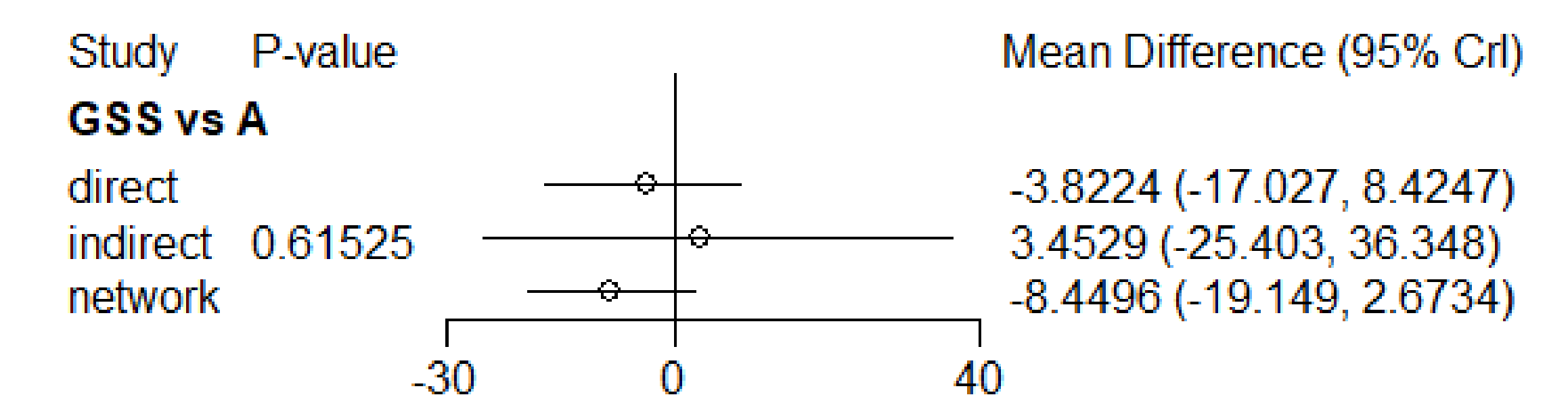
**

**Supplementary Figure 4 Node splitting diagram of WOMAC scores of stiffness**

**
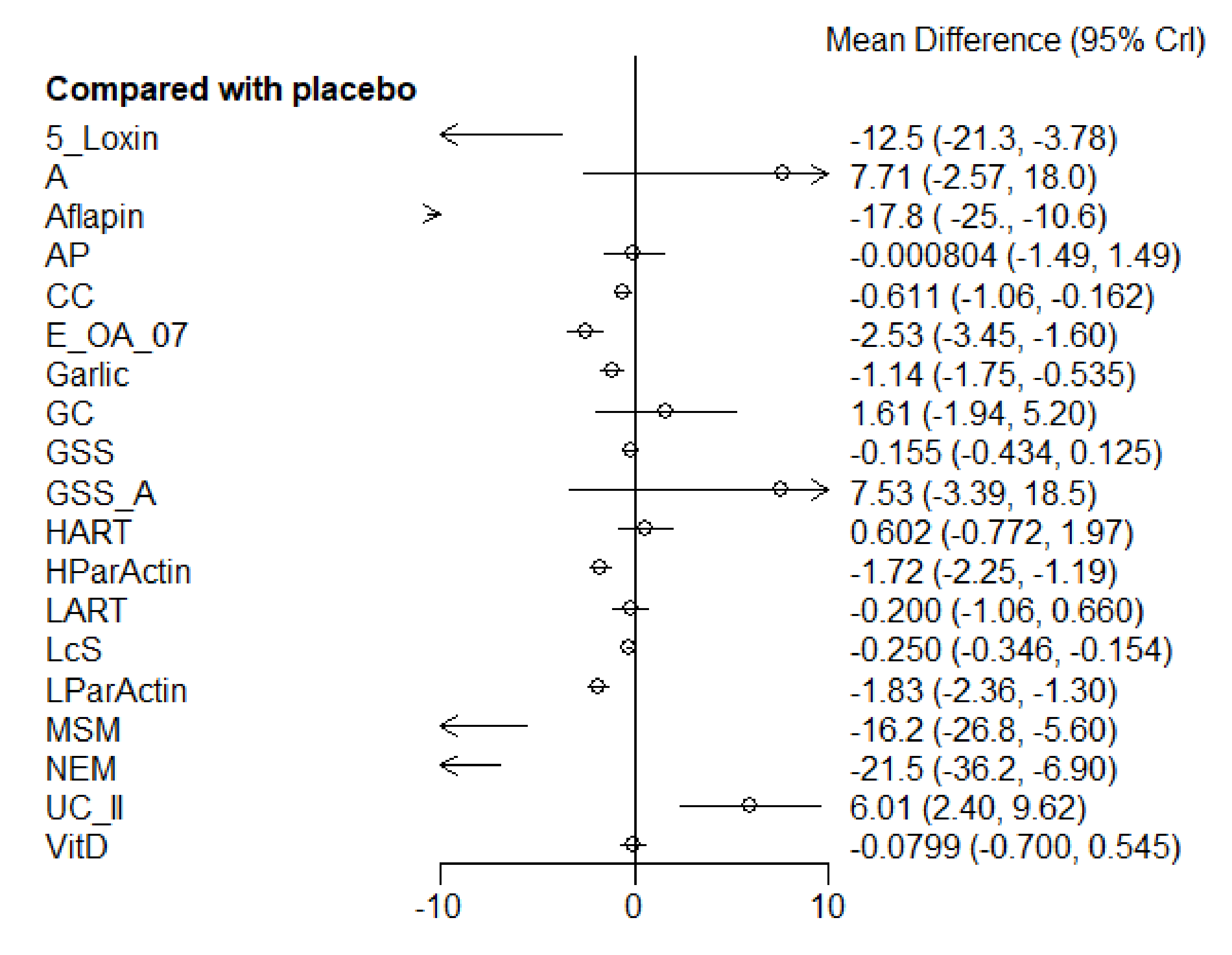
**

**Supplementary Figure 5 Forest plots of WOMAC scores of stiffness**

**
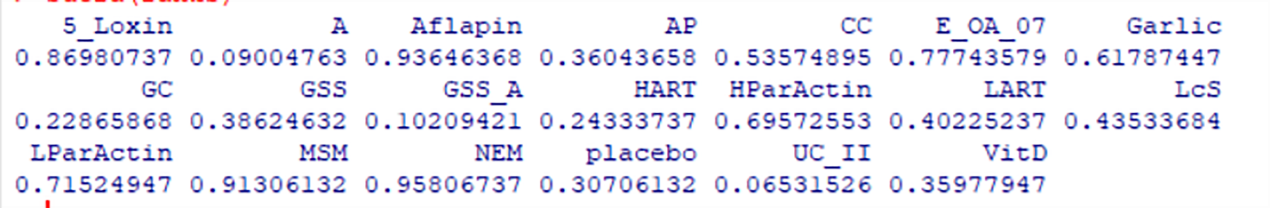
**

**Supplementary Figure 6 SUCRA of WOMAC scores of stiffness**

**
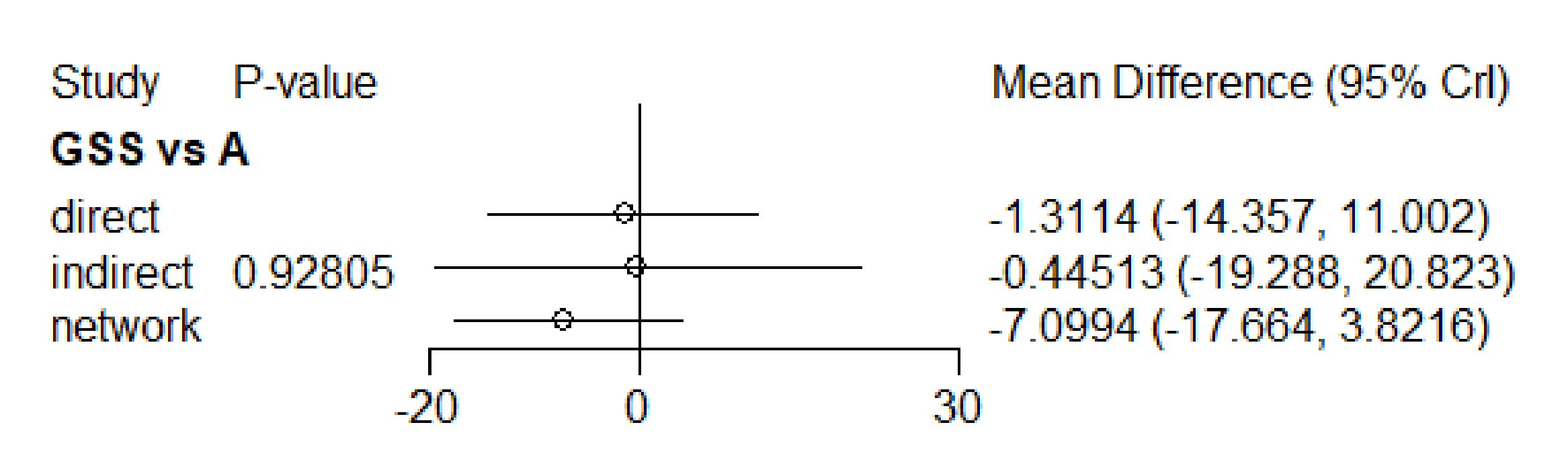
**

**Supplementary Figure 7 Node splitting diagram of WOMAC scores of pain**

**
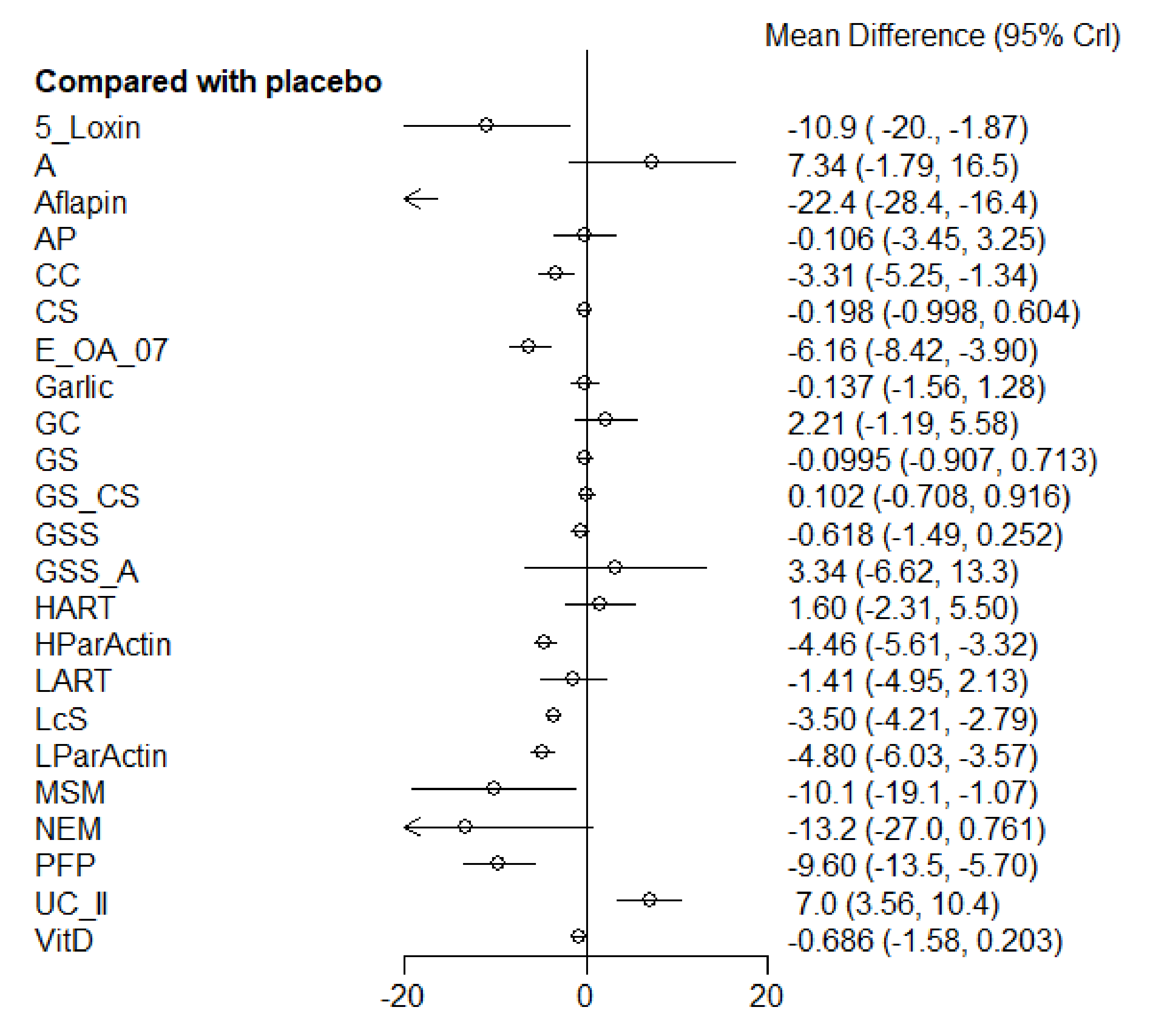
**

**Supplementary Figure 8 Forest plots of WOMAC scores of pain**

**
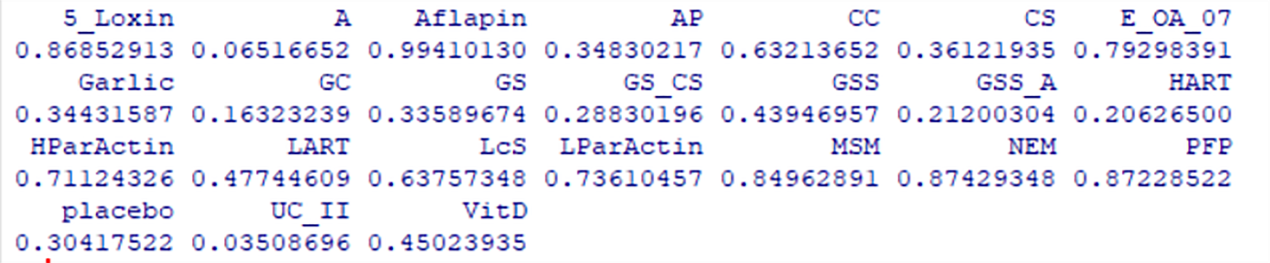
**

**Supplementary Figure 9 SUCRA of WOMAC scores of pain**

**
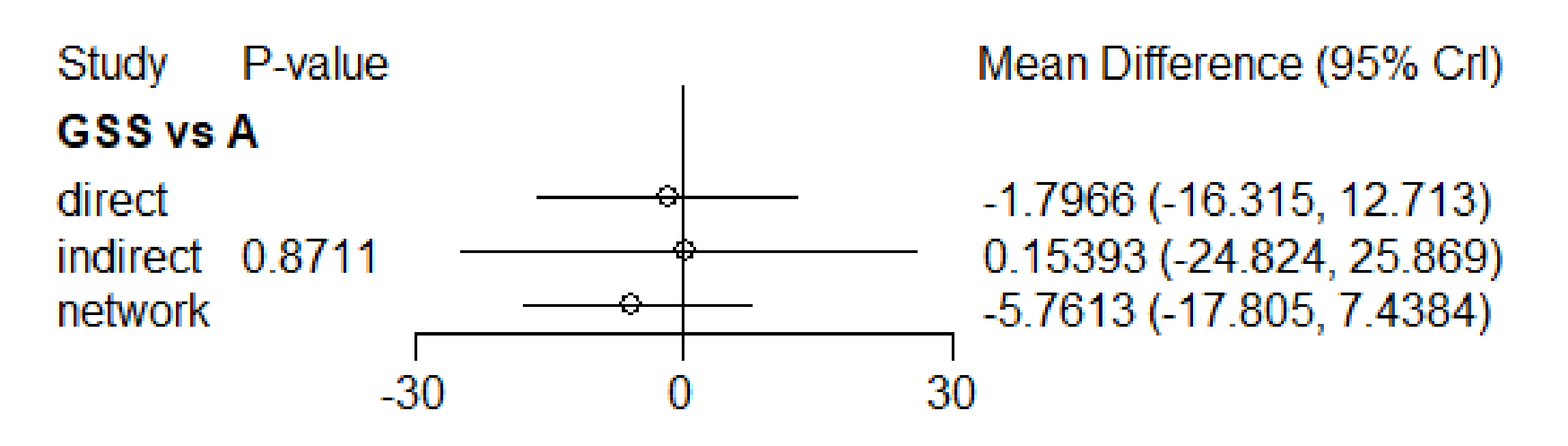
**

**Supplementary Figure 10 Node splitting diagram of WOMAC scores of function**

**
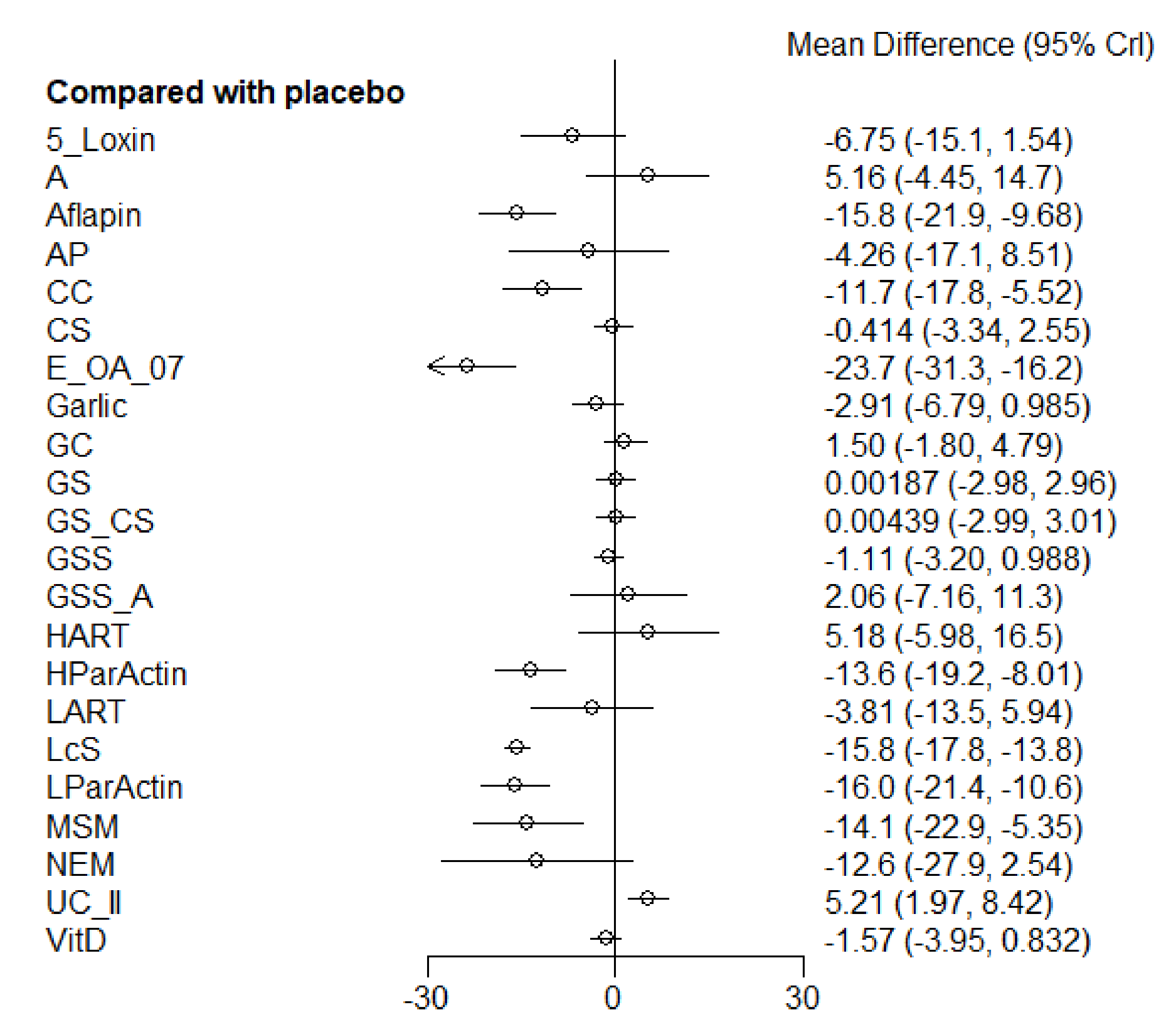
**

**Supplementary Figure 11 Forest plots of WOMAC scores of function**

**
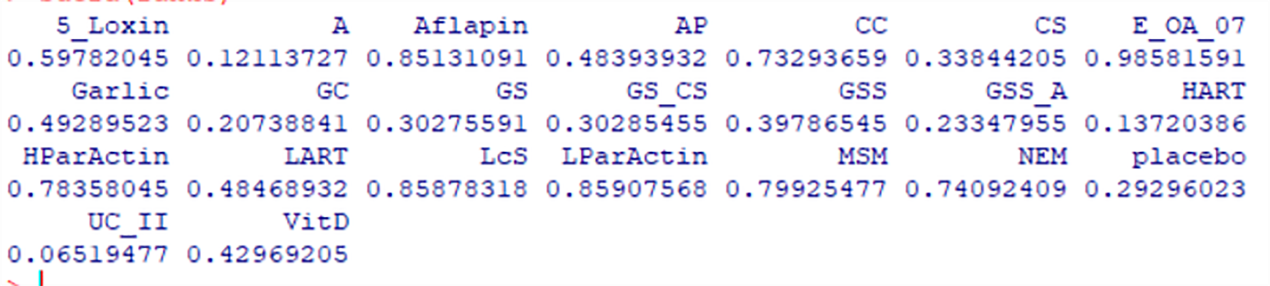
**

**Supplementary Figure 12 SUCRA of WOMAC scores of function**
